# FLT3L-induced virtual memory CD8 T cells engage the immune system against tumors

**DOI:** 10.1186/s12929-024-01006-9

**Published:** 2024-01-29

**Authors:** Hsin-Fang Tu, Yu-Jui Kung, Ling Lim, Julia Tao, Ming-Hung Hu, Michelle Cheng, Deyin Xing, T. C. Wu, Chien-Fu Hung

**Affiliations:** 1 Department of Pathology, Johns Hopkins University School of Medicine, 1550 Orleans Street, CRB II 307, Baltimore, MD 21287 USA; 2 Johns Hopkins University School of Medicine, Baltimore, MD USA; 3 Department of Biomedical Engineering, Johns Hopkins University, Baltimore, MD USA; 4 Department of Oncology, Johns Hopkins University School of Medicine, Baltimore, MD USA; 5 Department of Obstetrics and Gynecology, Johns Hopkins University School of Medicine, Baltimore, MD USA; 6 Molecular Microbiology and Immunology, Johns Hopkins University School of Medicine, Baltimore, MD USA

**Keywords:** CD44^high^ CD8 T cells, Virtual memory CD8 T cells, Alb-FLT3L

## Abstract

**Background:**

Previous research in FMS-like tyrosine kinase 3 ligands (FLT3L) has primarily focused on their potential to generate dendritic cells (DCs) from bone marrow progenitors, with a limited understanding of how these cells affect CD8 T cell function. In this study, we further investigated the in vivo role of FLT3L for the immunomodulatory capabilities of CD8 T cells.

**Methods:**

Albumin-conjugated FLT3L (Alb-FLT3L) was generated and applied for translational medicine purposes; here it was used to treat naïve C57BL/6 and OT1 mice for CD8 T cell response analysis. Syngeneic B16ova and E.G7ova mouse models were employed for adoptive cell transfer to evaluate the effects of Alb-FLT3L preconditioning of CD8 T cells on tumor progression. To uncover the underlying mechanisms of Alb-FLT3L modulation, we conducted bulk RNA-seq analysis of the CD44^high^ CD8 T cells. STAT1-deficient mice were used to elucidate the functional roles of Alb-FLT3L in the modulation of T cells. Finally, antibody blockade of type one interferon signaling and in vitro coculture of plasmacytoid DCs (pDCs) with naive CD8 T cells was performed to determine the role of pDCs in mediating regulation of CD44^high^ CD8 T cells.

**Results:**

CD44^high^ CD8 T cells were enhanced in C57BL/6 mice administrated with Alb-FLT3L. These CD8 T cells exhibited virtual memory features and had greater proliferative and effective functions. Notably, the adoptive transfer of CD44^high^ naïve CD8 T cells into C57BL/6 mice with B16ova tumors led to significant tumor regression. RNA-seq analysis of the CD44^high^ naïve CD8 T cells revealed FLT3L to induce CD44^high^ CD8 T cells in a JAK-STAT1 signaling pathway-dependent manner, as supported by results indicating a decreased ability of FLT3L to enhance CD8 T cell proliferation in STAT1-deficient mice as compared to wild-type control mice. Moreover, antibody blockade of type one interferon signaling restricted the generation of FLT3L-induced CD44^high^ CD8 T cells, while CD44 expression was able to be induced in naïve CD8 T cells cocultured with pDCs derived from FLT3L-treated mice. This suggests the crucial role of pDCs in mediating FLT3L regulation of CD44^high^ CD8 T cells.

**Conclusions:**

These findings provide critical insight and support the therapeutic potential of Alb-FLT3L as an immune modulator in preconditioning of naïve CD8 T cells for cancer immunotherapy.

**Supplementary Information:**

The online version contains supplementary material available at 10.1186/s12929-024-01006-9.

## Background

The development of immunotherapies has revolutionized the treatment of cancer over the past decades. Current immunotherapy approaches, including immune checkpoint inhibitors (ICIs), adoptive cell transfer (ACT), and CAR T cell therapy have shown promising clinical responses. However, their efficacies vary among patients and cancer types, and most patients do not benefit from these treatments [1]. This could be in part due to insufficient reprogramming of the immune suppressive tumor microenvironment, thus limiting the reinvigoration of antitumor immunity.

T cells with memory phenotype are equipped with superior antitumor functions and proliferative ability compared to effector T cells [2, 3]. Many promising immunotherapies have been centered on the expansion and regulation of memory T cells for anticancer purposes; however, the correlations between treatment responses and differentiation stages of memory T cells are still under debate [4–6]. In the past two decades, similar yet distinct subsets of CD8 T cells have been recognized to acquire memory features independently with antigen exposure [7]. For instance, virtual memory (VM) CD8 T cells were generated and maintained by expressions of transcription factor Eomesodermin (Eomes), type I IFN signaling, and IL-15 signaling [8–10]. These were first identified in germ-free mice with CD44^high^ memory phenotype [11, 12], and exhibit intermediate immune responses between naïve and true memory T cells, with unique functions of homeostatic proliferation and long-term maintenance in normal mice and humans [13–15]. Critically, despite their self-reactivity and memory phenotype features, VM CD8 T cells were found to be less likely to trigger autoimmune pathology than memory CD8 T cells [16]. Thus, the functionality of VM CD8 T cells in antitumor immunity is an important area for investigation.

Dendritic cells (DCs) are critical components of the immune system responsible for priming antigen-specific T cell responses in draining lymph nodes, recruiting activated T cells to tumor lesions, and enhancing the cytolytic activity of T cells. Hence, in addition to immunotherapies that aim to improve or re-direct T cell effector activity, current immunotherapies also shed light on methods to enhance DC functions. FMS-like tyrosine kinase 3 ligand (FLT3L) is an essential growth factor required for the hematopoietic progenitor cell development [17, 18], and its functions in expanding the numbers of plasmacytoid DC (pDC) and conventional DC (cDC1) subsets have been studied extensively [19, 20]. FLT3L-induced expansion of DCs in lymphoid and peripheral tissue has also been applied as an immunotherapeutic anticancer strategy in current preclinical and clinical studies [21, 22].

One challenge of using cytokine treatment, such as FLT3L, is its short serum half-life in humans [23]. Moreover, it is inefficient in penetrating secondary lymphoid tissues and tumor lesions [24, 25]. In the past few years, albumin conjugation has been considered one of the most effective strategies to extend the in vivo lifespans of peptides or protein-based molecules [26–30]. This is due to the biological nature of serum protein albumin, including its long half-life in vivo and its ability to act as a carrier protein. Using the albumin conjugation technique, our group successfully generated several immunomodulatory molecules previously, including Albumin (Alb)-GM-CSF, Alb-IL2, and Alb-IFNβ, for both vaccination and anti-cancer purposes [28–30]. In order to overcome the pertinent challenge of using FLT3L in treatment, we developed Alb-FLT3L by genetically fusing albumin with FLT3L. We demonstrated that Alb- FLT3L had a significantly extended half-life and increased accumulation in the lymph node and tumor. Alb- FLT3L was also shown to potentiate the expansion of dendritic cells and enhance T cell activation in tumor lesions, suggesting an immunomodulatory effect of Alb- FLT3L on CD8 T cells [31]. While FLT3L regulation of innate immune functions had been studied previously, its ability to improve T cell function had not been extensively evaluated, with only one study reporting FLT3L attenuation of T cell dysfunction and improvement in host resistance to burn wound sepsis in mice [32]. Given that CD8 T cells are critical players in anticancer immunity and their dysfunction contributes significantly to the immune-suppressive tumor microenvironment, we hope to further evaluate the important functional roles of Alb-FLT3L on CD8 T cells.

Herein, we highlight the potential of FLT3L in preconditioning CD8 T cell functions in vivo. Unexpectedly, we found a higher frequency of CD44^high^ naïve CD8 T cells in FLT3L-treated C57BL/6 mice compared to that in non-FLT3L-treated mice and discovered the underlying mechanism of FLT3L-induced CD44^high^ naïve CD8 T cells to be in a type I IFN signaling-dependent manner. These FLT3L-induced CD44^high^ naïve CD8 T cells were equipped with virtual memory T cell features and had greater proliferative and effective functions when activated. Furthermore, adoptive transfer of CD44^high^ naïve CD8 T cells into C57BL/6 mice with B16ova tumor led to tumor regression. Our data suggest that leveraging FLT3L preconditioning of naïve T cells may have significant potential in cancer immunotherapy.

## Material and methods

### Reagents and antibodies

For the generation of Alb-FLT3LL protein constructs, albumin was first amplified with PCR using cDNA template of albumin (NM_000477) purchased from transOMIC technologies (Huntsville, AL) and a set of primers, 5′-AAATCTAGAGCCACCATGAAGTGGGTAACCTTT-3′ and 5′-TTTGAATTCGGCTAAGGCGTCTTTGCATC-3′. The amplified product was then cloned into the XbaI/EcoRI sites of pcDNA3 vector (Invitrogen Corp., Carlsbad, California, USA).

Next, for the generation of pcDNA3-alb-FLT3L, FLT3 ligand was first PCR amplified using cDNA template of human FLT3 ligand (AAA90950.1) gene synthesized from Genscript (Piscataway, NJ) and a set of primers, 5′-TTTGAATTCACCCAGGACTGCTCCTTCCAA-3′ and 5′-AAAGGATCCTCACGAGGTCAGGAGATCGAG-3′. The amplified product was then cloned into the EcoRI/BamHI sites of pcDNA3-alb.

All plasmid constructs were confirmed by DNA sequencing. Albumin-FLT3L proteins were expressed using Expi293F expression system kit (Thermo Fisher Scientific, Waltham, MA) according to manufacturer’s instructions. Expi293F cells were transfected with pcDNA3-alb-FLT3L. Proteins were purified by HiTrap Albumin column (GE Healthcare Life Sciences, Marlborough, MA). Recombinant human FLT3L proteins were purchased from GeneScript (Piscataway, NJ).

The antibodies and reagents used in flow cytometry analysis are as follows:

**Table:** 

Antibodies	Catalog Number	Company
PE/Dazzle™ 594 anti-mouse/human CD44	103056	BioLegend
APC/Cyanine7 anti-mouse CD49d Antibody	103635	BioLegend
PE/Cyanine7 anti-mouse CCL5 (RANTES) Antibody	149105	BioLegend
PE anti-mouse CD5 Antibody	100607	Biolegend
PerCP/Cyanine5.5 anti-mouse Ly-6C Antibody	128011	BioLegend
FITC anti-mouse CD314 (NKG2D) Antibody	115711	BioLegend
Alexa Fluor® 647 anti-mouse EOMES Antibody	157703	Biolegend
PE/Cyanine5 anti-mouse CD69 Antibody	104510	Biolegend
APC anti-mouse IFN-γ Antibody	505810	Biolegend
Brilliant Violet 421™ anti-mouse Ki-67 Antibody	652411	Biolegend
APC/Fire™ 750 anti-mouse CD8a Antibody	100766	BioLegend
Zombie AquaTM Fixable Viability Kit	423102	Biolegend
Brilliant Violet 650™ anti-mouse CD8a Antibody	100742	Biolegend
Brilliant Violet 785™ anti-mouse CD3 Antibody	100231	Biolegend
APC/Fire™ 750 anti-mouse CD3 Antibody	100247	Biolegend
Brilliant Violet 421™ anti-mouse CD11c Antibody	117330	Biolegend
PE/Cyanine7 anti-mouse I-A/I-E Antibody	107630	Biolegend
PE/Dazzle™ 594 anti-mouse/human CD44 Antibody	103056	Biolegend
CD44 Monoclonal Antibody (IM7), PE	12-0441-82	eBioscience™
FITC Anti-CD8 alpha antibody [KT15]	Ab22504	abcam
T-Select H-2Kb OVA Tetramer-SIINFEKL-PE	TB-5001-M	MBL

### Ethics approval

All animals were housed and handled in the animal facility of the Johns Hopkins Medical Institution under specific-pathogen-free conditions. All procedures were performed according to approved protocols by the Johns Hopkins Medical Institutions Animal Care and Use Committee and the National Institutes of Health.

### Mice

6-week-old female C57BL/6 mice were purchased from Taconic Biosciences (Germantown, NY). OT-1, and STAT1^−/−^ mice of the C57BL/6 background were purchased from Jackson Laboratories (Farmington, CT). All mice were maintained at the Johns Hopkins University School of Medicine (Baltimore, MD) animal facility under specific pathogen-free conditions.

### Cell culture

Mouse melanoma B16-OVA cells were acquired as a generous gift from Dr. Charles Drake and maintained in complete DMEM media supplemented with 10% FBS, 1% l-glutamine, 100 U/ml penicillin, 100 mg/ml streptomycin, 2 mM l-glutamine, 2 mM sodium pyruvate, and 2 mM non-essential amino acid.

### In vitro CD44^high^ T cell activation and proliferation

Lymphocytes were taken from the spleen and lymph nodes of C57BL/6, OT-1, and STAT1−/− mice with or without FLT3L treatment. RBCs were lysed by the addition of excess RBC lysis buffer twice (Cell Signaling Technology, Danvers, Massachusetts) followed by extensive washing in 0.5% BSA/PBS (FACS buffer). Cells were stained with PE-conjugated CD44 antibodies in the staining buffer, washed, and resuspended in the staining buffer. To assess the functions of CD44high CD8 T cells, CD44-positive cells were isolated using the EasySep™ PE Positive Selection Kit II (Stem Cell Technologies). The isolation efficiency was confirmed by flow cytometry analysis. For the T cell proliferation assay, lymphocytes (1 × 10^6^ cells/ml) were suspended in phosphate-buffered saline (PBS) and labeled with the CellTrace Violet™ (Thermo Fisher Scientific, Waltham, MA) at a concentration of 10 μM at 37 °C for 20 min. After labeling, an excess of 10% RPMI/FBS was added to the samples to quench the reaction. Following centrifugation and extensive washing, cells were resuspended in 200 ml of 10% RPMI/FBS with T cell stimulant: either α-CD3 (1.25 ug/ml) and α-CD28 (0.25 ug/ml) antibodies for lymphocytes from C57BL/6 mice, or OVA257-264 (SIINFEKL, 10 or 50 ng/ml) peptides for lymphocytes from OT-1 mice for 24 to 48 h. Cells were then harvested for analysis by flow cytometry.

### Flow cytometry and cell sorting

Blood samples were obtained from the mouse facial vein by lancet and collected into EDTA-coated tubes. Lymphocytes harvested from the lymph nodes or spleen were prepared in single-cell suspensions. RBCs were lysed by the addition of excess RBC lysis buffer twice (Cell Signaling Technology, Danvers, Massachusetts) followed by extensive washing in 0.5%BSA/PBS (FACS buffer). Single staining controls of ultracomp ebeads (Thermo Fisher Scientific, Waltham, MA) were used to set a compensation matrix for each experiment. FMO and isotype staining controls were used to set gating and determine non-specific binding. Before antibody staining, Zombie Aqua live/dead (BioLegend, San Diego, CA) was used for dead cell discrimination according to manufacturer instructions. Fc Block was used prior to antibody staining. Antibody and tetramer dilutions were determined through titration. All antibody mixes were stained for at least 30 min at 4 degrees Celsius. Data collection was done on a 13-color Beckman Coulter CytoFLEX S, and analysis was done with FlowJo 10.4 software (FlowJo LLC). BD FACSAria™ Fusion sorter was used for cell sorting at the Johns Hopkins School of Medicine CRB HP Flow Core facility.

### Intracellular staining

For intranuclear staining to measure cytokine production, mouse lymphocytes were treated with protein transport inhibitor Brefeldin A (BioLegend, San Diego, CA) for 16 h. Cells were then collected, and prepared as described in the flow cytometry section. At the end of extracellular staining, cells were permeabilized using eBioscience Foxp3/Transcription Factor Staining Buffer Set (Thermo Fisher Scientific, Waltham, MA) and stained for intracellular cytokines.

### Adoptive cell transfer

Lymphocytes were collected from the spleen and lymph nodes of C57BL/6 mice with or without administration of FLT3L treatment, and single-cell suspensions were prepared. RBCs were lysed by the addition of excess RBC lysis buffer twice (Cell Signaling Technology, Danvers, Massachusetts) followed by extensive washing in 0.5% BSA/PBS (FACS buffer). Cells were stained with fluorescence-conjugated CD3, CD8, and CD44 antibodies in the staining buffer, washed, and then resuspended in the staining buffer. The CD44^high^ CD8 T cells were sorted by flow cytometry for adoptive transfer via retro-orbital injection.

### Bulk RNA-sequencing analysis

For bulk RNA-sequencing, CD44^high^ CD8 T cells preparation followed the same procedure as described for adoptive cell transfer. Total RNA was extracted with RNeasy Mini Kit (Qiagen, Hilden, Germany) according to manufacturer instruction. The preparation of the RNA library and transcriptome sequencing was conducted by Novogene Co., LTD (Beijing, China). Genes with adjusted p-value < 0.05 and |log2(Fold Change) |> 0 were considered as differentially expressed.

### Tumor experiments

For the tumor experiments, 2 × 10^5^ B16-ova cells were inoculated subcutaneously in the abdomen of each C57BL/6 mouse. Tumor growth was monitored by digital caliper reading and the size was measured and calculated using the formula *length* * *length* * *width* * *0.5*. The endpoints of the experiment were determined once the diameter of tumors surpassed 2 cm or when any loss in body weight was observed as per our approved animal protocols. Tumors and draining lymph nodes were harvested for flow cytometry analysis. For tumor infiltration leukocyte analysis, tissue was collected from mice and placed in FACS buffer with magnesium and calcium. After mincing with scissors into ~ 2 mm pieces, tissue digestion enzymes including Collagenase I, Collagenase IV, and DNase I were added to samples and incubated for 20 min at 37 degrees Celsius. Excess 10% RPMI/FBS was then added to the samples to quench the reaction. Following centrifugation, samples were filtered using a 70-mm cell strainer. Then, tumor samples were purified by Ficoll gradient. Cells were resuspended in 10% RPMI/FBS and loaded onto Ficoll-Paque Plus (GE Healthcare Life Sciences, Marlborough, MA) under a centrifugation condition of 400×*g* at 25 °C for 30 min. The lymphocyte layer was harvested and washed twice with phosphate-buffered saline solution (PBS), followed by centrifugation at 400×*g* at 25 °C for 10 min. Samples were then counted, plated at equal cell numbers, and prepared for flow cytometry. The draining lymph nodes were dissociated using a syringe plunger and 70 uM filter and washed using FACS buffer. Cell suspensions were then RBC lysed, washed, and resuspended in FACS buffer for downstream analysis.

### In vitro dendritic cell-T cell coculture assay

For plasmacytoid (pDC) enrichment, splenocytes were harvested from FLT3L-treated C57BL/6 mice and pDC were isolated according to the manufacturer’s instructions using the EasySep™ Mouse Plasmacytoid DC Isolation Kit (STEMCELL, Vancouver, Canada). After isolation, pDC cells were resuspended in complete RPMI media supplemented with 10% FBS, 1% l-glutamine, 100 U/ml penicillin, 100 mg/ml streptomycin, 2 mM l-glutamine, 2 mM sodium pyruvate, 2 mM non-essential amino acid, and 50 µM 2β-mercaptoethanol. For FLT3L-preconditioned BMDCs preparation, bone marrow was collected from the femurs of C57BL/6 mice after euthanasia. Bone marrow cells were isolated by flushing femurs with complete RPMI media. RBCs were lysed by the addition of excess RBC lysis buffer twice (Cell Signaling Technology, Danvers, Massachusetts) followed by extensive washing in complete RPMI media. Cells were then resuspended in complete RPMI and seeded at 1 × 10^6 ^cells/ml in a 6-well plate with 20 ng/ml recombinant GM-CSF (Peprotech, Waltham, MA) or 200 ng/ml recombinant human FLT3L (GeneScript, Piscataway, NJ) for 7 days. On day 3, cells received an additional 2 ml of fresh complete RPMI with recombinant GM-CSF or FLT3L proteins. On Day 7, culture media was removed and centrifuged at 300×*g* for 5 min to recover non-adherent cells. After centrifugation, the supernatant was aspirated, and the BMDCs were resuspended in complete RPMI media.

For naïve CD8 T cell isolation, lymphocytes were harvested from the lymph nodes and spleen, and processed into single-cell suspensions. RBCs were lysed as previously described. Naïve CD8 T cells were enriched according to the manufacturer’s instructions using the EasySep™ Mouse Naïve CD8^+^ T Cell Isolation Kit (STEMCELL, Vancouver, Canada). After isolation, T cells were resuspended in complete RPMI media. For coculture experiments, different ratios of pDCs or FLT3L-preconditioned BMDCs were incubated with 2 × 10^5^ naïve CD8 T cells in 96-well round-bottom tissue culture plates. After 3 days of coculture, cells were collected and stained with zombie Aqua live/dead (BioLegend, San Diego, CA), CD3, CD8α, and CD44 for flow cytometry analysis.

### Statistical analysis

All data are expressed as means ± standard error of the mean (S.E.M). The statistical significance was determined by one-way ANOVA with Tukey–Kramer multiple comparison or Student’s t-test using Prism 9 software (GraphPad, CA, USA). In all circumstances, *p*-values ≤ 0.05 were considered significant (*, *p* < 0.05; **, *p* < 0.01; ***, *p* < 0.001; ****,* p* < 0.0001).

## Results

### FLT3L preconditions naïve CD8 T cells to memory-like CD8 T cells

**Fig. 1:**
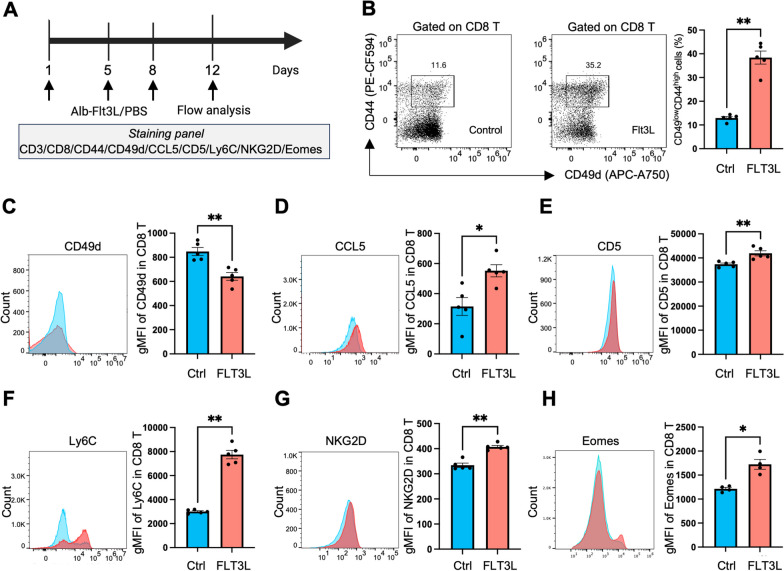
FLT3L modulated Naïve CD8 T cells display memory-like phenotype. **A** C57BL/6 mice were administered either Alb-FLT3L (100 ug) or vehicle control on days 1, 5, and 8 for a total of 3 doses. PBMCs collected on Day 12 after initiation of treatment were assessed for virtual-memory phenotype expressions by flow cytometry. **B** Representative gating of CD44 and CD49d expressions on CD8 T cells. The frequencies of CD49d^low^ CD44^high^ CD8 T cells for the indicated treatment groups. **C**–**H** The mean fluorescent intensity of CD49d (**C**), CCL5 (**D**), CD5 (**E**), Ly6C (**F**), NKG2D (**G**), and Eomes (**H**) in CD8 T cells for the indicated treatment groups. N = 5 mice per group. Data is represented by mean ± SEM. P values were calculated by ordinary one-way ANOVA with the Tukey–Kramer multiple comparison test, and P < 0.05 is considered statistically significant. *, **, ***, and ****Indicate p values less than 0.05, 0.01, 0.001, and 0.0001, respectively

FLT3L has been shown to be functional in the expansion of DCs, yet its effects through DCs on CD8 T cell differentiation remain unknown. To address this issue, we analyzed the differentiation of T cell subsets in the peripheral blood of 6-week-old female C57BL/6J mice treated with Alb-FLT3L (Fig. 1A, Additional file 1: Figure S1A), and found high frequencies of CD44^high^ CD8 T cells with central memory (CD44^high^CD62L^high^) and effector (CD44^high^CD62L^low^) phenotypes following treatment (Additional file 1: Figure S1B–D). In contrast, there were no significant differences in the differentiation of CD4 T cells under treatment (Additional file 1: Figure S1E, F). To determine whether FLT3L-induced CD44^high^ CD8 T cells were VM T cells, which can be distinguished from true memory T cells through their lowered CD49d expression, we analyzed CD8 T cells in the peripheral blood and found that FLT3L-treated mice had higher frequencies of CD49d^low^CD44^high^ CD8 T cells (Fig. 1B), and significantly lower mean fluorescent intensities (MFI) of CD49d compared to control mice (Fig. 1C). Furthermore, we examined markers that have been reported to be presented in virtual memory T cells [9, 10, 33–36], including CCL5, CD5, Ly6C, NKG2D, Eomes, CXCR3, and CD122; and found MFI expressions of all the featured proteins to be increased in the CD8 T cells of FLT3L-treated mice (Fig. 1D–H, Additional file 1: Figures S2, S3B, C).We utilized UMAP plots to visualize the distribution of various proteins associated with virtual memory T cells (Cd49d, CCL5, CD5, Ly6C, and NKG2D), as well as CD44 and CD62L within the CD8 T cell populations obtained from a control mouse and an Alb-FLT3L-treated mouse. A distinct region of the cells exhibiting the VM phenotype was identified, which was notably heightened in the Alb-FLT3L-treated mice (Additional file 1: Figure S4). Furthermore, there were no obvious changes in the T cell activation marker (CD25) and exhaustion markers (PD1, CTLA4, and Lag3) pre- versus post-treatment with Alb-FLT3L, confirming the distinctness of FLT3L-induced CD44^high^ CD8 T cells from effector cells (Additional file 1: Figures S5, S6). Of note, a comparison of FLT3L and Alb-FLT3L proteins in inducing CD49d^low^CD44^high^ CD8 T cells in mice suggested that both FLT3L and Alb-FLT3L could induce VM CD8 T cell populations. However, Alb-FLT3L more efficiently induced the VM CD8 T cell population compared to treatment with FLT3L (Additional file 1: Figure S7). The results indicate an immunomodulatory effect of Alb-FLT3L on inducing a VM phenotype in naïve CD8 T cells.

### FLT3L-induced CD44^high^ CD8 T cells exhibit greater activation and effector functions

**Fig. 2:**
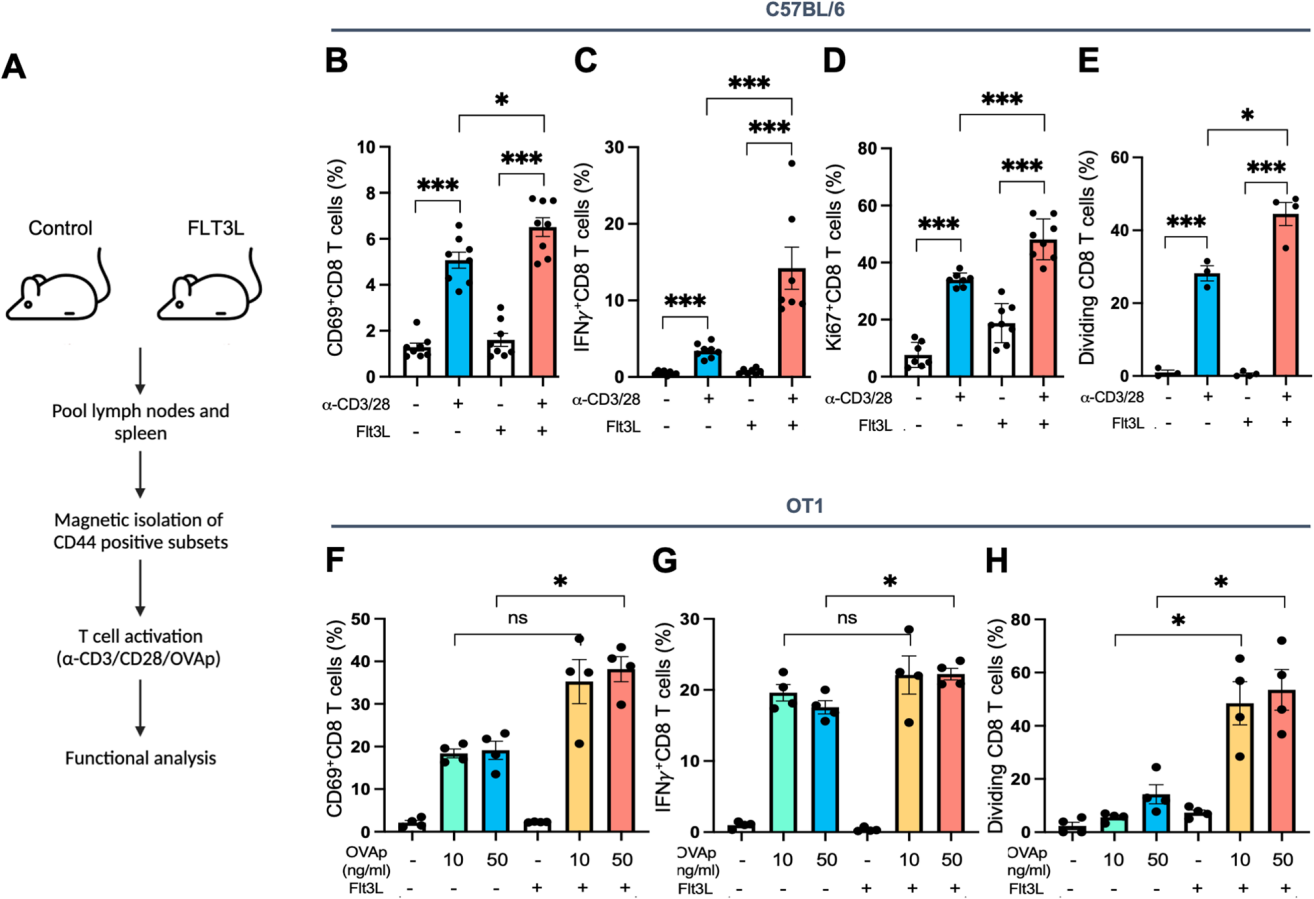
FLT3L-induced CD44^high^ CD8 T cells exhibit greater proliferative and effector functions. **A** For the assessment of T-cell activation and effector function in Alb-FLT3Linduced CD44^high^ CD8 T cells on Day 12 after initial treatment, control and Alb-FLT3L treated mice were euthanized, and CD44 subsets were magnetically enriched from pooled lymphocytes and splenocytes. T cells were stimulated for 24 to 48 h with either CD3 and CD28 antibodies for C57BL/6 mice, or OVA257-264 (SIINFEKL) peptides for OT-1 mice. **B**–**D** The frequency of CD69^+^ (**B**), IFNγ^+^ (**C**), and Ki67^+^ (**D**) CD8 T cells in C57BL/6 mice for the indicated treatment group. **E** The frequencies of dividing cells in C57BL/6 mice for the indicated treatment group. **F**–**H** The frequency of CD69^+^ (**F**), IFNγ^+^ (**G**), and dividing (**H**) CD8 T cells in OT-1 mice for the indicated treatment group. Data is represented by mean ± SEM. p values were calculated by ordinary one-way ANOVA with the Tukey–Kramer multiple comparison test, and p < 0.05 is considered statistically significant. *, **, ***, and ****Indicate p values less than 0.05, 0.01, 0.001, and 0.0001, respectively

To examine functional differences between FLT3L-induced and vehicle control CD44^high^ CD8 T cells, lymphocytes were individually harvested from the lymph nodes and spleen of control and FLT3L-treated OT1 mice. CD44^high^ CD8 T cells were isolated by magnetic beads and stained with Cell Trace Violet™, followed by T-cell activation using anti-CD3 and anti-CD28 antibodies (Fig. 2A). FLT3L-induced CD44^high^ CD8 T cells demonstrated improved capabilities for T cell activation (Fig. 2B, Additional file 1: Figure S8A), effector function (Fig. 2C, Additional file 1: Figure S8B), and proliferation (Fig. 2D, E, Additional file 1: Figure S8C, D). Additionally, to investigate whether FLT3L can modulate antigen-specific CD8 T cell immune responses, CD44^high^ CD8 T cells were collected from OT-1 mice administrated with FLT3L treatment and stimulated with ova peptides (257–264) for CD8 T cell activation. These results similarly revealed the ability of FLT3L to enhance antigen-specific T-cell activation (Fig. 2F, Additional file 1: Figure S8E), effector function (Fig. 2G, Additional file 1: Figure S8F), and proliferation (Fig. 2H, Additional file 1: Figure S8G). Notably, a low dose (10 ng/ml) of ova peptide stimulation was capable of significantly enhancing CD8 T cell proliferation (Fig. 2H), potentially implying FLT3L ability to lower the immune threshold and decrease the amount of antigen needed for T-cell activation and proliferation.

### Preconditioning of CD44^+^CD8 T cells by FLT3L improves anti-tumor immunity

**Fig. 3:**
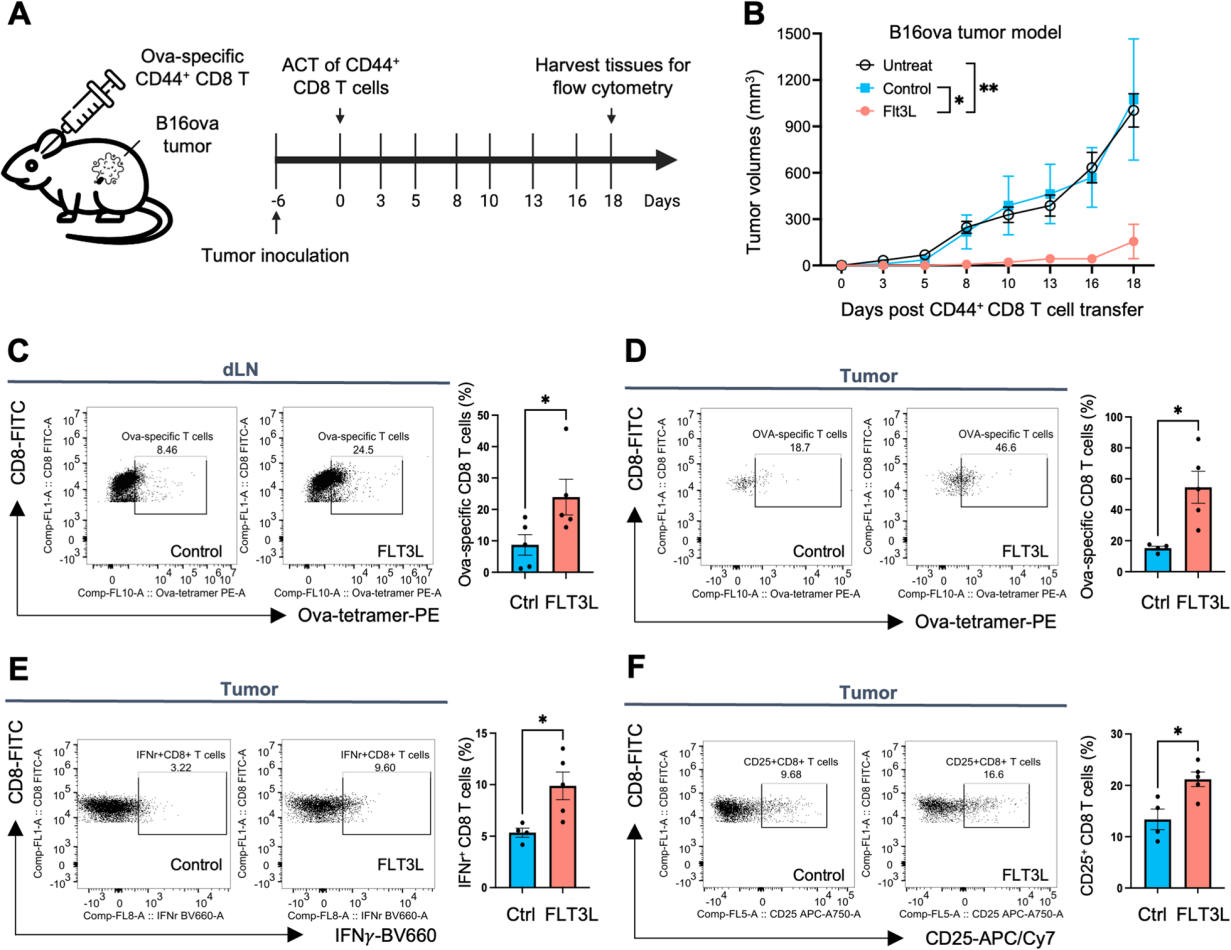
FLT3L-induced CD44^high^ CD8 T cells elicit tumor immunity against B16ova tumors. **A** C57BL/6 mice were inoculated with B16-OVA cells subcutaneously. Six days later, mice were injected intravenously through the retroorbital sinus with 2 × 10^6^ CD44^high^ CD8 T cells that were sorted from control naïve or FLT3L-treated OT-1 mice. Eighteen days after adoptive T cell implantation, mice were euthanized and tumor tissues and drained lymph nodes were harvested for flow cytometry analysis. **B** Tumor growth curve following the described treatment protocol. **C**, **D** The frequencies of OVA-specific CD8 T cells in the tumor-draining lymph node (**C**) and tumor (**D**). **E** The frequencies of IFNγ^+^ CD8 T cells in the tumors **F**. The frequencies of CD25^+^ CD8 T cells in the tumors. N = 5 mice per group. Data is represented by mean ± SEM. p values were calculated by ordinary one-way ANOVA with the Tukey–Kramer multiple comparison test, and p < 0.05 is considered statistically significant. *, **, ***, and ****Indicate p values less than 0.05, 0.01, 0.001, and 0.0001, respectively

To determine whether FLT3L-preconditioned CD44^high^ CD8 T cells could provide better control of tumors, we performed adoptive cell transfer (ACT) therapies in poorly immunogenic Ova-expressing B16 and E.G7 syngeneic tumor models (Fig. 3A). Ova-specific CD44^high^ CD8 T cells isolated from the FLT3L-treated OT-1 mice (Additional file 1: Figure S9) were delivered to either B16-ova or E.G7-ova -bearing mice via retro-orbital injection. The mice that received ACT of CD44^high^ CD8 T cells from FLT3L-treated OT-1 mice showed tumor regression, while untreated mice and those that received ACT from vehicle control mice did not (Fig. 3B, Additional file 1: Figure S10). We used ova-tetramer staining to trace the CD44^high^ CD8 T cells distribution from donor mice and found the ACT of FLT3L-preconditioned CD44^high^ CD8 T cells resulted in higher tetramer-specific CD8 T cell infiltration in tumor-draining lymph nodes and tumor lesions (Fig. 3C, D). Expressions of inflammatory cytokine (IFNγ) and T lymphocyte activation (CD25) were also examined in the tumor-infiltrating CD8 T cells, and were significantly increased in mice receiving ACT of CD44^high^ CD8 T cells from the FLT3L-treated OT-1 mice (Fig. 3E, F, Additional file 1: Figure S11). Moreover, immunophenotyping of lymphoid and myeloid populations in treated tumors revealed an increase in CD4^+^ T cells, CD8^+^ T cells, and natural killer (NK) cells, respectively (Additional file 1: Figure S12A, D, G). In contrast, we did not observe differences in populations including Tregs, B cells, CD11c^+^ DCs, MDSCs, and macrophages (Additional file 1: Figure S12C, H–K). Further characterization of infiltrating CD8 T cells revealed increased numbers of CD25^+^ and IFNγ^+^ CD8^+^ T cells in mice receiving adoptive transfer of FLT3L-preconditioned CD44^high^ CD8 T cells compared to transfer from vehicle control mice (Additional file 1: Figure S12E, F). These results suggest that adoptive transfer of FLT3L-preconditioned CD44^high^ CD8 T cells could modify the tumor microenvironment, promoting a hot tumor in the poorly immunogenic B16-OVA tumor model, and leading to enhanced tumor clearance.

### JAK-STAT signaling participates in FLT3L-modulated function of CD44^high^ CD8 T cells

**Fig. 4:**
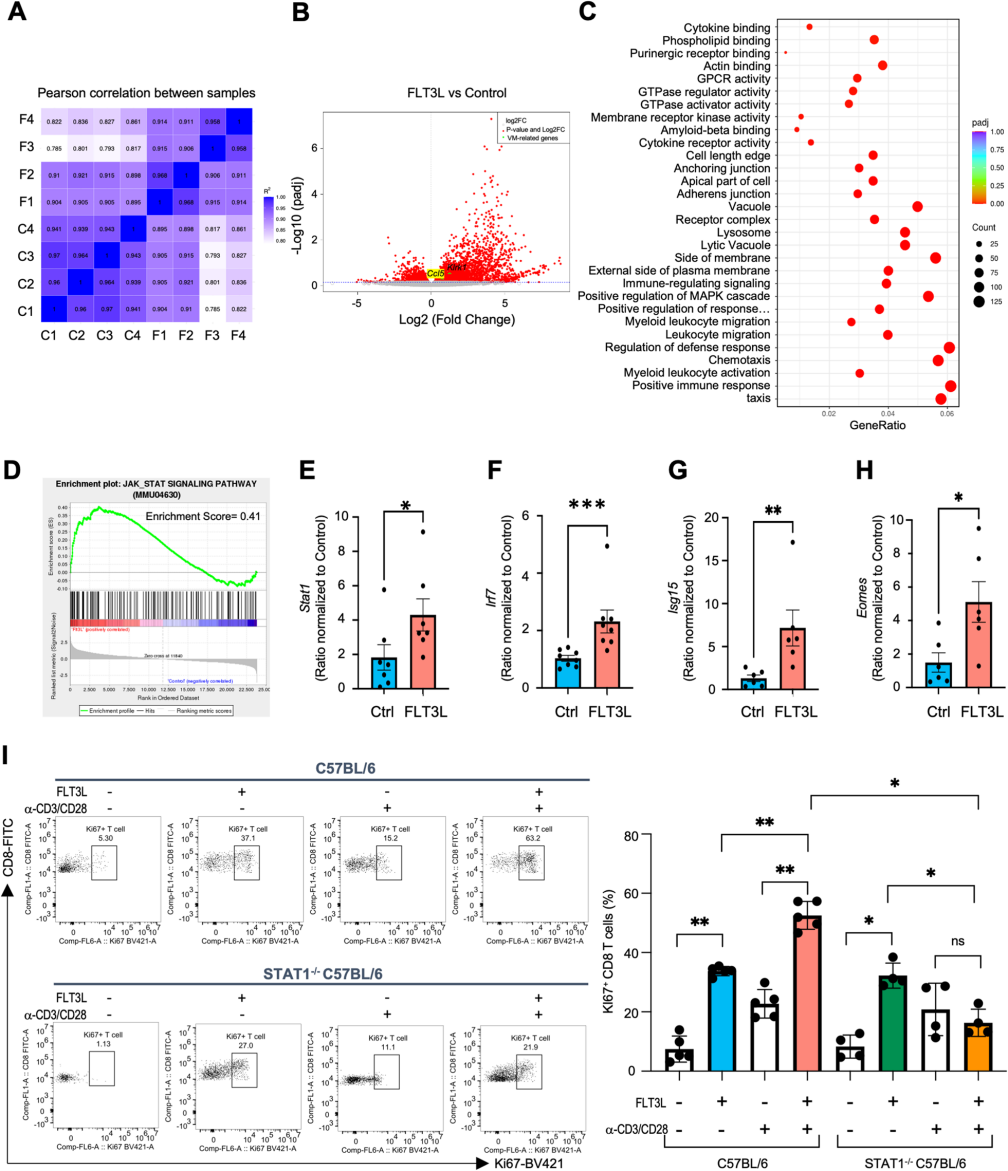
FLT3L-induced CD44^high^ CD8 T cells elevate genes involved in the JAK-STAT1 signaling pathway. For bulk RNA-seq analysis, CD44^high^ CD8 T cells were sorted from control naïve or FLT3L-treated C57BL/6 mice. **A** Pearson correlation analysis between all samples. **B** Volcano plot of RNA-seq transcriptome data displaying the pattern of gene expression values for FLT3L-induced CD44^high^ CD8 T cells relative to vehicle control CD44^high^ CD8 T cells. Significantly differentially expressed genes (FDR-corrected p ≤ 0.05) were highlighted in red. VM-related genes, including *Ccl5* and *Klrl1* were highlighted in green. **C**, **D** Enrichment plots from gene set enrichment analysis (GSEA) displaying enriched biological processes, molecular functions, and cellular components associated with genes upregulated in FLT3L-induced relative to vehicle control CD44^high^ CD8 T cells. **E**–**H** qPCR analysis confirmed gene expressions associated with the JAK-STAT pathway. **I** CD44-positive immune subsets were enriched from control and Alb-FLT3L treated B6-wild type (WT) or B6-STAT1^−/−^ mice. After cell enrichment, cells were stimulated with CD3 and CD28 antibodies for 72 h. The frequency of Ki67+ CD8 T cells in the indicated treatment group. Data is represented by mean ± SEM. p values were calculated by ordinary one-way ANOVA with the Tukey–Kramer multiple comparison test, and p < 0.05 is considered statistically significant. *,**,***, and ****Indicate p values less than 0.05, 0.01, 0.001, and 0.0001, respectively

To gain biological insight into the molecular response to FLT3L in CD44^high^ CD8 T cells, we performed RNA-seq analysis of flow-sorted CD44^high^ CD8 T cells from the FLT3L-treated and vehicle control C57BL/6J mice. Pearson correlation analysis, principal component analysis (PCA), and the hierarchical clustering heatmap revealed the inter- and intragroup variability between samples (Fig. 4A, Additional file 1: Figure S13). DESeq2 standard workflow was employed to explore the differentially expressed genes between the FLT3L-treated and control groups. The analysis identified 2090 up-regulated and 464 down-regulated genes in the FLT3L-treated group compared to control groups (Fig. 4B). The up-regulated differential genes were significantly enriched in the positive regulation of immune response, chemotaxis, and cytokine-cytokine receptor interaction (Fig. 4C, Additional file 1: Figure S14A). These upregulated genes were involved in inflammatory chemokine receptors and regulation of T cell activation and effector functions, including CCR2 and Stx7, suggesting the CD44^high^ CD8 T cells derived from FLT3L-treated group had enhanced effector potential compared to control groups (Additional file 1: Figure S14B). Furthermore, we found a positive correlation of the JAK-STAT signaling pathway in FLT3L-preconditioned CD44^high^ CD8 T cells via a gene set enrichment analysis (GSEA)-based pathway analysis (Fig. 4D). Validating RNA-seq data by qPCR analysis confirmed that genes involved in the JAK-STAT signaling pathway were highly expressed in FLT3L-preconditioned CD44^high^ CD8 T cells (Fig. 4E–H). We also applied a STAT1 knockout (C57BL/6-STAT1^−/−^) mouse model to investigate the significance of STAT1 in regulating the functions of the CD44^high^ CD8 T cells. Comparison of CD44^high^ CD8 T cell induction by FLT3L between wild-type and STAT1 knockout mice indicated that FLT3L enhancement of cell proliferation was dampened in STAT1 knockout mice, even with FLT3L administration Fig. 4I). The results suggest that the JAK-STAT signaling pathway plays a critical role in the functional maintenance of CD44^high^ CD8 T cells.

### DCs are key mediators of FLT3L to drive CD8 T cells toward a virtual-memory phenotype

**Fig. 5:**
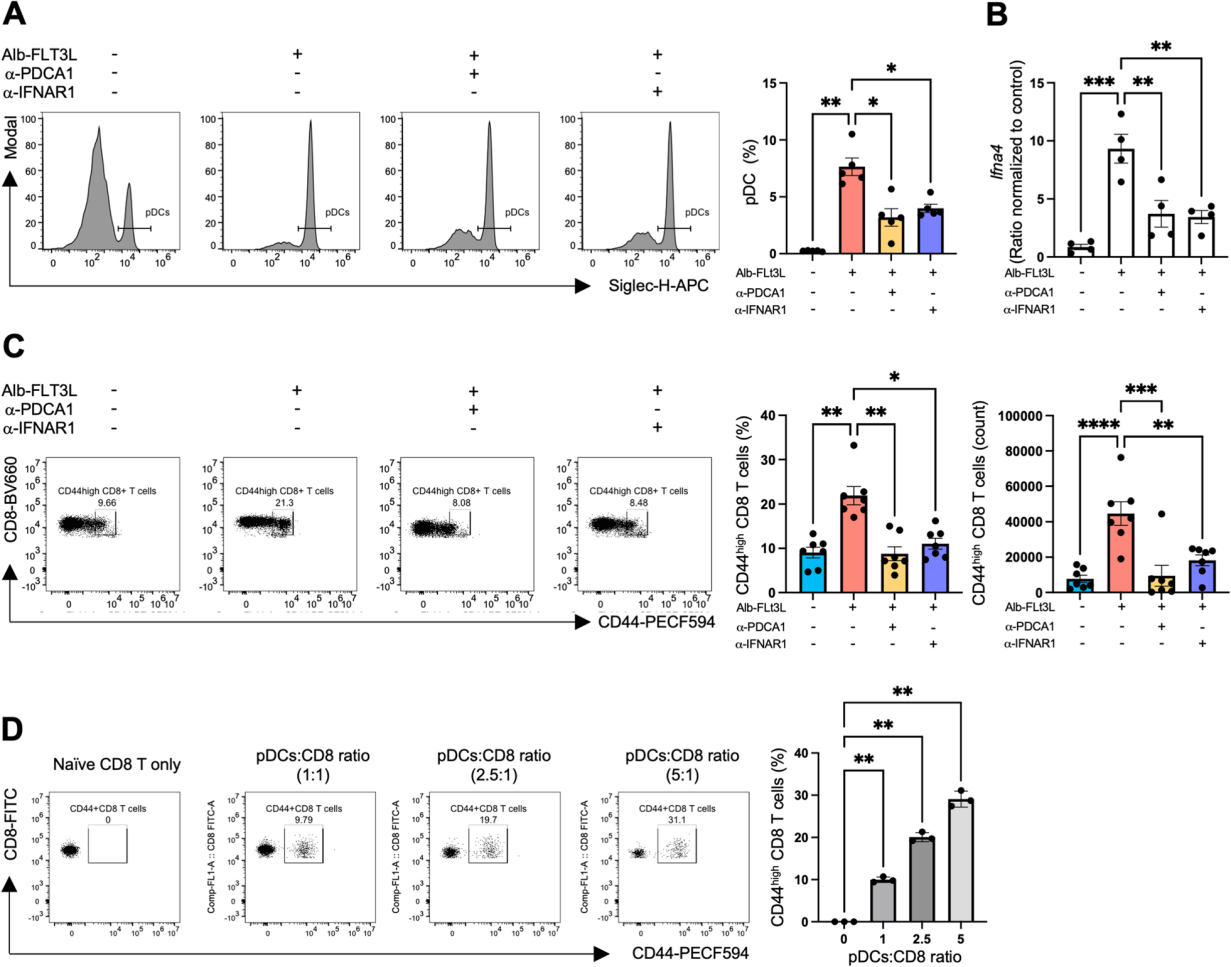
Type I IFNs are important for the preconditioning of CD44^high^ CD8 T cells. For the evaluation of type I IFN signaling affecting FLT3L in the preconditioning of CD44^high^ CD8 T cells, antibody blockade targeting either PDCA1 or IFNAR1 was used before and between the Alb-FLT3L treatment intervals. Mice were treated with 200 ug αPDCA or 500 ug aIFNAR1. Specifically, antibody blockade was performed on Day 1, 2, 4, 5, 8, 9 and 11. Mice were treated with 50 ug Alb-FLT3L on Day 3, 7 and 10. On Day 13, peripheral blood and spleen samples were collected and processed for flow cytometry and qPCR analysis. **A** Representative figures of pDCs (CD11C^int^MHC2^low^Siglec-H^+^). The Modal option was used in Flowjo to normalize each peak to its mode. The frequencies of pDC in the indicated treatment group. **B** qPCR analysis confirmed *Ifna4* expression levels. **C** Frequencies and numbers of CD44^high^ CD8 T cells. **D** Naïve CD8 T cells were cocultured with increasing ratios of pDCs for 3 days. The frequencies of CD44^high^ CD8 T cells in the indicated groups. Data is represented by mean ± SEM. p values were calculated by ordinary one-way ANOVA with the Tukey–Kramer multiple comparison test, and p < 0.05 is considered statistically significant. *, **, ***, and ****Indicate p values less than 0.05, 0.01, 0.001, and 0.0001, respectively

Type one interferons (type I IFNs) are cytokines that play a central role in modulating antiviral immune responses and controlling crosstalk between innate and adaptive immune responses [37]. Canonically, type I IFNs activate the JAK-STAT pathway by binding to the IFN-α/β receptors, leading to the transcription of IFN-stimulated genes (ISGs) [38]. FLT3 receptors are predominantly expressed on myeloid dendritic cells (mDCs) and plasmacytoid dendritic cells (pDCs), less expressed in classical monocytes and eosinophils, and rarely expressed in T cells (Additional file 1: Figure S15A). To rule out the direct effects of FLT3L on CD8 T cells, naïve CD8 T cells were isolated from B6 mice and incubated with varying concentrations of FLT3L for 3 and 6 days. The findings showed FLT3L had marginal effects on inducing CD44 expressions in the CD8 T cells (Additional file 1: Figure S15B). Given that pDCs are the most potent IFN-producing cells [39], we hypothesized that the actions of FLT3L on inducing CD44^high^ CD8 T cells might be mediated by type I IFN stimulation from pDCs. To confirm this hypothesis, either anti-IFNAR1 antibodies (to block type one interferon signaling) or anti-PDCA-1 antibodies (to deplete pDCs) were administered to C57BL/6 mice prior to FLT3L treatment. The frequencies of pDC in the spleen were verified by flow cytometry (Fig. 5A, Additional file 1: Figure S17), and IFNA4 gene expression in splenocytes under FLT3L and type I signaling blockade treatment was confirmed by qPCR analysis (Fig. 5B). The results implied that either inhibition of type I IFN signaling or depletion of pDC could significantly dampen the ability of FLT3L to precondition CD44^high^ CD8 T cells (Fig. 5C). Furthermore, to demonstrate the direct effects of pDC in inducing VM CD8 T cell phenotype, magnetically enriched pDC from the spleen of FLT3L-treated mice (Additional file 1: Figure S16A) or FLT3L-derived BMDCs (Additional file 1: Figure S15C, D) were cocultured with naïve CD8 T cells (Additional file 1: Figure S16B). After 3 days of co-culture, CD44 expression levels in CD8 T cells were found to be elevated with increasing ratios of pDC or FLT3L-derived BMDCs (Fig. 5F, Additional file 1: Figure S15E). These results suggest that type I IFNs from pDC are critical in the modulation of CD44^high^ CD8 T cells.

## Discussion

**Fig. 6:**
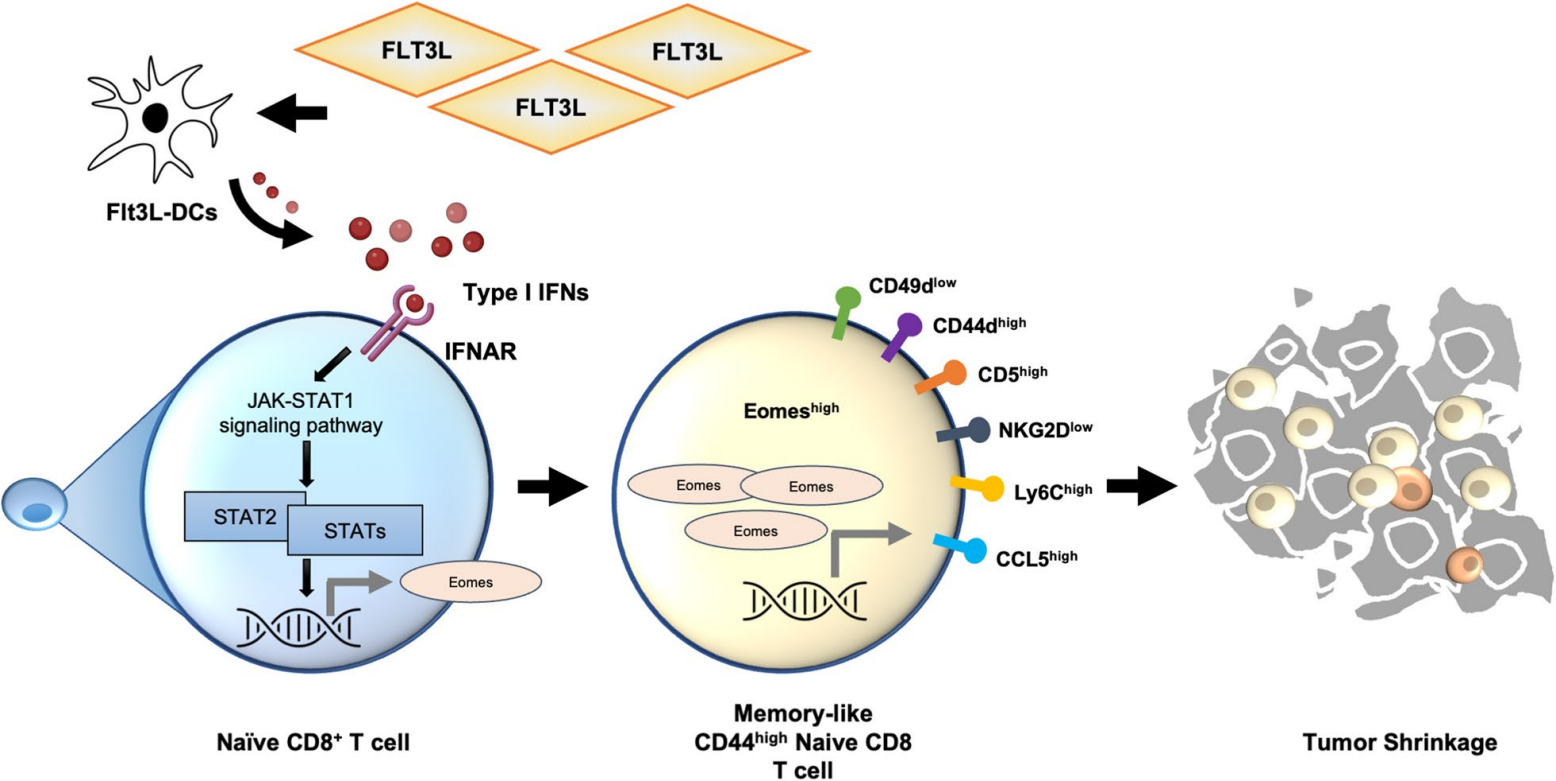
Schematic summary of this study. In this study, we demonstrated the role of FLT3L in regulating naïve CD8 T cells toward a memory-like phenotype. The FLT3L-induced CD44^high^ naïve CD8 T cells were equipped with virtual memory T cell features and had greater proliferative and effective functions against solid tumors. The underlying mechanism was led through DCs in a type I IFN signaling-dependent manner. Our data provide new insights into FLT3L for the preconditioning of naïve T cells in cancer immunotherapy

This research is among the first to discuss the bystander activation of naïve CD8 T cells driven by FLT3L to elicit protective immunity. Our study demonstrates the crucial role FLT3L plays in driving naïve CD8 T cells towards a memory-like phenotype. These cells show superior proliferative and effector functions against tumors, with an underlying mechanism dependent on type I IFN signaling. Our data may ultimately give new understanding into cancer immunotherapy that utilizes FLT3L preconditioning of naive T cells (Fig. 6).

VM CD8 T cells have been considered a natural consequence of heterogeneity in a naïve CD8 T cell pool, yet there is currently little understanding of the origin and development of these cells. Most studies believe VM CD8 T cells equipped with high self-ligand affinity are derived from autoreactive T cells during thymus selection and are further matured in the periphery [8, 40]. Previous studies have recognized that the differentiation of VM CD8 T cells is regulated by Eomes expression, the intrinsic factor in immature single-positive CD8 thymocytes. This expression is further regulated by cytokine regulation, including type 1 IFN and IL-15. Nevertheless, an investigation into which specific signaling regulates the differentiation process is still needed. This could be done by exploring the contribution of unique cytokines or growth factors to VM CD8 T cell differentiation in the fetal stage or in adults. It is known that IL-15 is a critical cytokine for the development and maintenance of VM T cells in mice [12, 41]. Furthermore, FLT3L-induced of CD44^high^ CD8 T cells have been shown to express IL-15Rβ (CD122 or IL2Rβ) and Eomes, a T-box transcription factor that promotes IL-15Rβ expression [42]. Hence, it is worth investigating the effects of IL-15 on the functionality and cell proliferation of FLT3L-induced of CD44^high^ CD8 T cells. Our results showed consistency with previous findings in VM CD8 T cell development and provided further understanding in this field of research. Whether manipulation of FLT3L can aid in the therapeutic treatment of disease warrants further investigation in the future.

In the H-2Kb OVA tetramer staining, we recognize that the staining results in Fig. 3C do not align with the anticipated distinct positive population typically observed with OT-I CD8 T cells. In our tetramer staining approach, we initially conducted antibody titration to determine the appropriate tetramer dilutions for cell staining. Nonetheless, several factors could potentially influence the quality of antibody staining, with inadequate antibody titration being a significant concern that may result in suboptimal staining outcomes. Furthermore, while we excluded dead cells through live/dead staining, the presence of damaged cells originating from the tumor or draining lymph nodes might still hinder the effectiveness of tetramer binding.

STAT1-KO (C57BL/6-STAT1^−/−^) mice are known to be a useful mouse model for studying the mechanisms of IFN-induced and STAT1-dependant resistance to infection and disease. We adopted this mouse model to verify that FLT3L-primed CD8 T cells were regulated through the JAK-STAT pathway. More explicit experiments could also be considered, including conditional gene knockout mice models, drug inhibition on particular pathways, or adoptive cell transfer experiments to gain a more conclusive mechanism of FLT3L in preconditioning naïve CD8 T cells.

The FLT3/FLT3L axis is pivotal in the maturation and differentiation of multiple immune cells [43–46]. Because of its multidimensional roles, FLT3L has been extensively studied for its therapeutic potential [47–50]. A combination therapy of FLT3L with α-PD1 blockade resulted in significantly reduced tumor growth and increased survival in preclinical and clinical trials [22, 51]. FLT3L-expressing CAR-T therapy improved tumor clearance with an increase in endogenous DC and T cells [52]. FLT3L also expanded the DC population and potentiated cancer vaccine immune responses in patients with metastatic melanoma [53]. Administration of FLT3L generates VM T cells, which may raise concerns about autoimmune issues. Nevertheless, compared to checkpoint blockade treatment which commonly induce immune-related adverse effects (irAE) and intolerable side effects, the side effects from FLT3L treatment were manageable and tolerable [54]. In our animal study, we observed no specific side effects in mice that underwent Alb-FLT3L treatment, such as weight loss (Additional file 1: Figure S18A), hair loss, or color changes. Furthermore, H&E staining of vital organs such as the heart, lungs, liver, kidney, and pancreas did not indicate significant immune cell infiltration in the tissues of Alb-FLT3L-treated mice (Additional file 1: Figure S18B). Our results, along with previous research, show that administration of FLT3L is less likely to cause severe autoimmune diseases. Additionally, this study provides new insights into FLT3L's anti-tumor effects by modulating CD8 T cells with VM features during adoptive cell transfer treatment.

In this study, we utilized various markers to distinguish FLT3L-preconditioned CD8 T cells from conventional naïve T cells or memory T cells. T memory stem cells (T_SCM_ cells) are recognized for their ability to self-renew and generate TCM, TEM, and Teff cells. There is a rising interest in exploring the similarities between FLT3L-preconditioned CD8 T cells and Tscm cells, as well as investigating the potential of FLT3L to induce this specific subset of T cells. The inclusion of more in-depth transcriptomic analysis will be helpful in characterizing the heterogeneity of these T-cell populations.

## Conclusions

This study is informative in elucidating how naïve CD8 T cells respond to growth factor FLT3L via DCs in adult mice. We demonstrated that FLT3L-induced CD8 T cells possess better immune functions and contribute to antitumor immune responses. Our findings indicate that applications of FLT3L could include T-cell preconditioning treatments for anticancer purposes, or in combination with a vaccine to boost efficacy. More clinical evidence is still awaited to support the findings here.

### Supplementary Information

**Additional file 1: Figure S1.** Phenotypical analysis of T cell differentiation in C57BL/6J mice receiving Alb-FLT3L treatment. **Figure S2.** Gating strategies and representative figures were shown in Fig. 1C–H. **Figure S3.** Expressions of Cd62L, CXCR3, and CD122 in CD8 T cells in C57BL/6J mice receiving Alb-FLT3L treatment. **Figure S4.** UMAP projection of Flow cytometry data from both a vehicle control and an Alb-Flt3L treated mouse. **Figure S5.** FLT3L-induced VM CD8 T cells were not been activated. **Figure S6.** Expressions of exhaustion markers in VM CD8 T cells. **Figure S7.** Frequencies of CD49lowCD44highCD8 T cells in C57BL/6J mice receiving FLT3L and Alb-FLT3L treatment. **Figure S8.** Representative figures for flow cytometry were shown in Fig. 2. **Figure S9.** The FACS sorting strategy of CD44high CD8 T cells from control naïve or FLT3L-treated OT1 mice. **Figure S10.** FLT3L-induced CD44high CD8 T cells elicit tumor immunity against E.G7-ova tumors. **Figure S11.** Gating strategies and representative figures for flow cytometry were shown in Fig. 3C–F. **Figure S12.** Tumor immunophenotype analysis in B16ova tumor-bearing mice received adoptive cell transfer of FLT3L-preconditioned CD44high CD8 T cells. **Figure S13.** Bulk RNA-seq analysis of CD44high CD8 T cells between FLT3L and control groups. **Figure S14.** Differential gene expression analysis of bulk RNA-seq data. **Figure S15.** pDCs are critical mediators of FLT3L to drive CD8 T cells toward a virtual-memory phenotype. **Figure S16.** Magnetic isolation efficiency of pDC and naïve CD8 T cells. **Figure S17.** Gating strategies and representative figures of pDC for flow cytometry were shown in Fig. 5A. **Figure S18.** Body weight and H&E staining of major organs in C57BL/6 mice received Alb-FLT3L treatment.

## Data Availability

All data relevant to the study are included in the article or uploaded as supplementary information. Data and materials are available on reasonable request.
