# Supplementary material for: FLT3L-induced virtual memory CD8 T cells engage the immune system against tumors
Source: J Biomed Sci. 2024 Jan 29;31:19. doi: 10.1186/s12929-024-01006-9 (PMC10826030; doi:10.1186/s12929-024-01006-9)
Supplement: Supplementary file 1 — Additional file 1: Figure S1. Phenotypical analysis of T cell differentiation in C57BL/6J mice receiving Alb-FLT3L treatment. Figure S2. Gating strategies and representative figures were shown in Fig. 1C–H. Figure S3. Expressions of Cd62L, CXCR3, and CD122 in CD8 T cells in C57BL/6J mice receiving Alb-FLT3L treatment. Figure S4. UMAP projection of Flow cytometry data from both a vehicle control and an Alb-Flt3L treated mouse. Figure S5. FLT3L-induced VM CD8 T cells were not been activated. Figure S6. Expressions of exhaustion markers in VM CD8 T cells. Figure S7. Frequencies of CD49lowCD44highCD8 T cells in C57BL/6J mice receiving FLT3L and Alb-FLT3L treatment. Figure S8. Representative figures for flow cytometry were shown in Fig. 2. Figure S9. The FACS sorting strategy of CD44high CD8 T cells from control naïve or FLT3L-treated OT1 mice. Figure S10. FLT3L-induced CD44high CD8 T cells elicit tumor immunity against E.G7-ova tumors. Figure S11. Gating strategies and representative figures for flow cytometry were shown in Fig. 3C–F. Figure S12. Tumor immunophenotype analysis in B16ova tumor-bearing mice received adoptive cell transfer of FLT3L-preconditioned CD44high CD8 T cells. Figure S13. Bulk RNA-seq analysis of CD44high CD8 T cells between FLT3L and control groups. Figure S14. Differential gene expression analysis of bulk RNA-seq data. Figure S15. pDCs are critical mediators of FLT3L to drive CD8 T cells toward a virtual-memory phenotype. Figure S16. Magnetic isolation efficiency of pDC and naïve CD8 T cells. Figure S17. Gating strategies and representative figures of pDC for flow cytometry were shown in Fig. 5A. Figure S18. Body weight and H&E staining of major organs in C57BL/6 mice received Alb-FLT3L treatment. [file 12929_2024_1006_MOESM1_ESM.pdf]

Additional file for *FLT3L-induced Virtual Memory CD8 T cells Engage the Immune System Against Tumors*

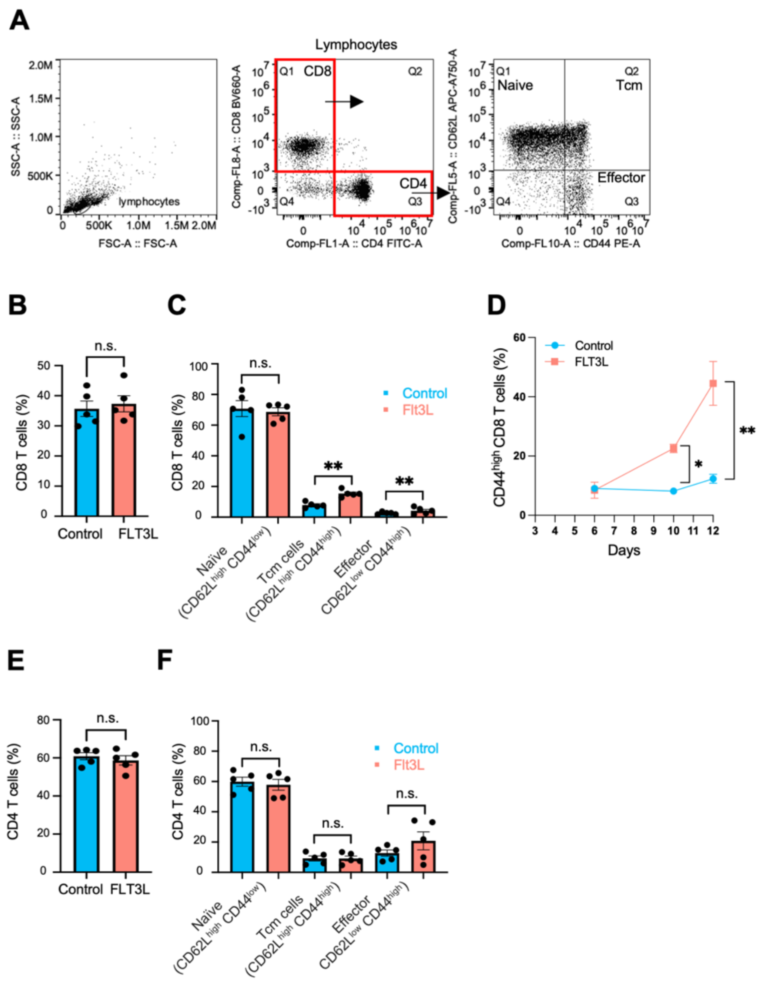

Figure S1. Phenotypical analysis of T cell differentiation in C57BL/6J mice receiving Alb-FLT3L treatment. C57BL/6 mice were administered either Alb-Flt3L (100 µg) or vehicle control on days 1, 5, and 8 for a total of 3 doses. PBMCs collected on Day 6, Day 10 and Day 12 after initiation of treatment were assessed by flow cytometry. (A) Schematic representative of gating strategy for T cell differentiation in CD4 and CD8 T cells. (B-C) Frequencies of naive (Naive, $CD44^{low}CD62L^{high}$), central memory (Tcm; $CD44^{high}CD62L^{high}$), and effectors (Effector; $CD44^{high}CD62L^{low}$) populations in CD8 T cells on Day 12 after Alb-Flt3L treatment. (D) Frequencies of $CD44^{high}$CD8 T cells on different time points in indicated groups. (E-F) Frequencies of naive (Naive, $CD44^{low}CD62L^{high}$), central memory (Tcm; $CD44^{high}CD62L^{high}$), and effectors (Effector; $CD44^{high}CD62L^{low}$) populations in CD4 T cells on Day 12 after Alb-Flt3L treatment.

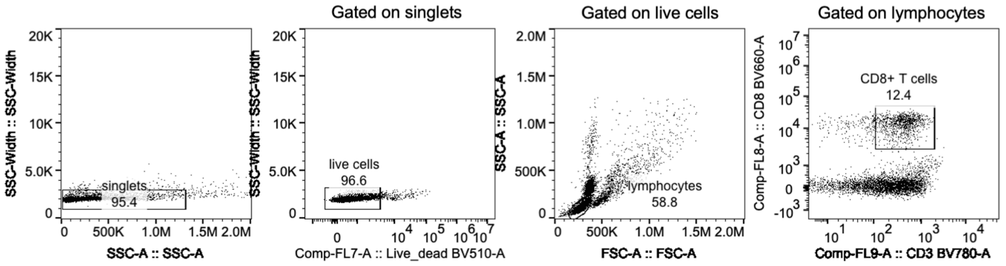

Figure S2. Gating strategies and representative figures were shown in Figure 1C-1H.

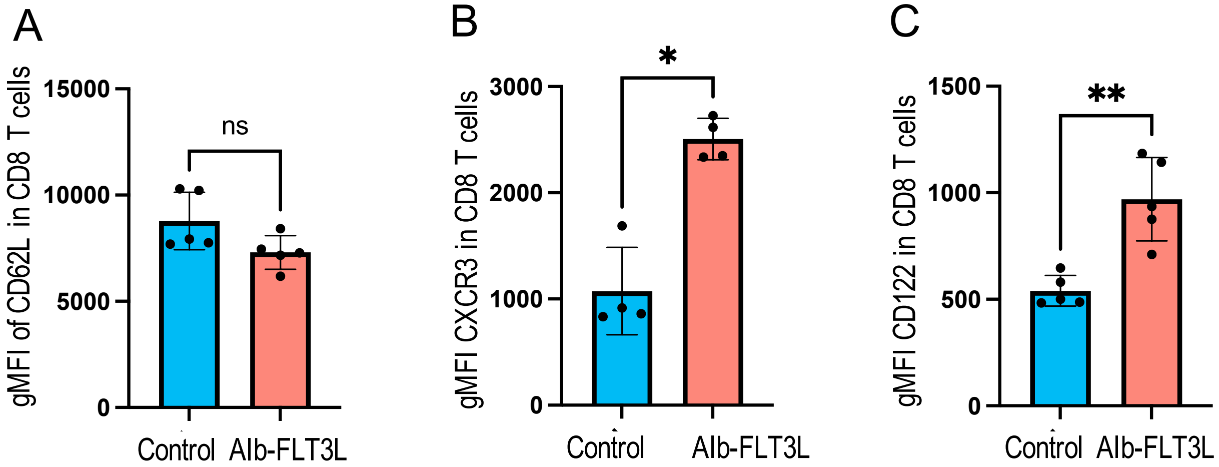

Figure S3. Expressions of Cd62L, CXCR3, and CD122 in CD8 T cells in C57BL/6J mice receiving Alb-FLT3L treatment. C57BL/6 mice were administered either Alb-Flt3L (100 μg) or vehicle control on days 1, 5, and 8 for a total of 3 doses. PBMCs collected on Day 12 after initiation of treatment were assessed by flow cytometry. Geometric mean fluorescence intensity (gMFI) of CD62L (A), CXCR3 (B), and CD122 (C) in CD8 T cells obtained from indicated treatment groups.

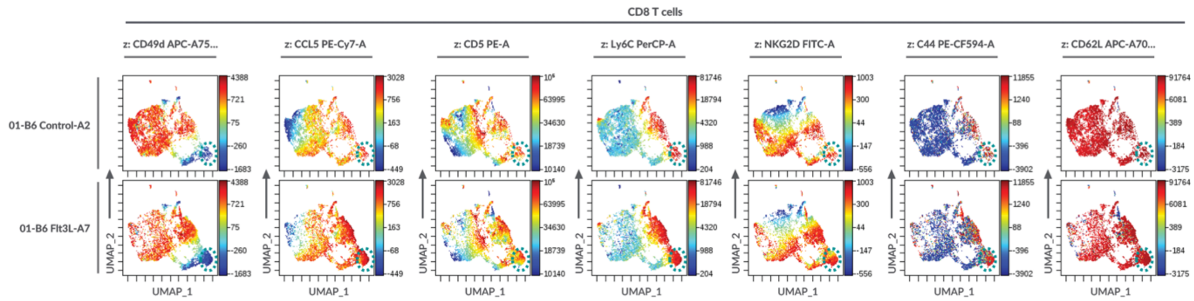

Figure S4. UMAP projection of Flow cytometry data from both a vehicle control and an Alb-Flt3L treated mouse. C57BL/6 mice were administered either Alb-Flt3L (100 mg) or a vehicle control on days 1, 5, and 8, totaling three doses. Peripheral blood mononuclear cells (PBMCs) were collected on Day 6, Day 10, and Day 12 after the initiation of treatment and assessed using an 8-marker flow cytometry panel. FCS files were uploaded to Cytobank, a cloud-based single-cell analysis software. CD3+CD8 live cells were gated prior to dimensionality reduction. UMAP plots were generated with cells color-coded according to the expression intensity of CD49d, CCL5, CD5, Ly6C, NKG2D, CD44, and CD62L individually.

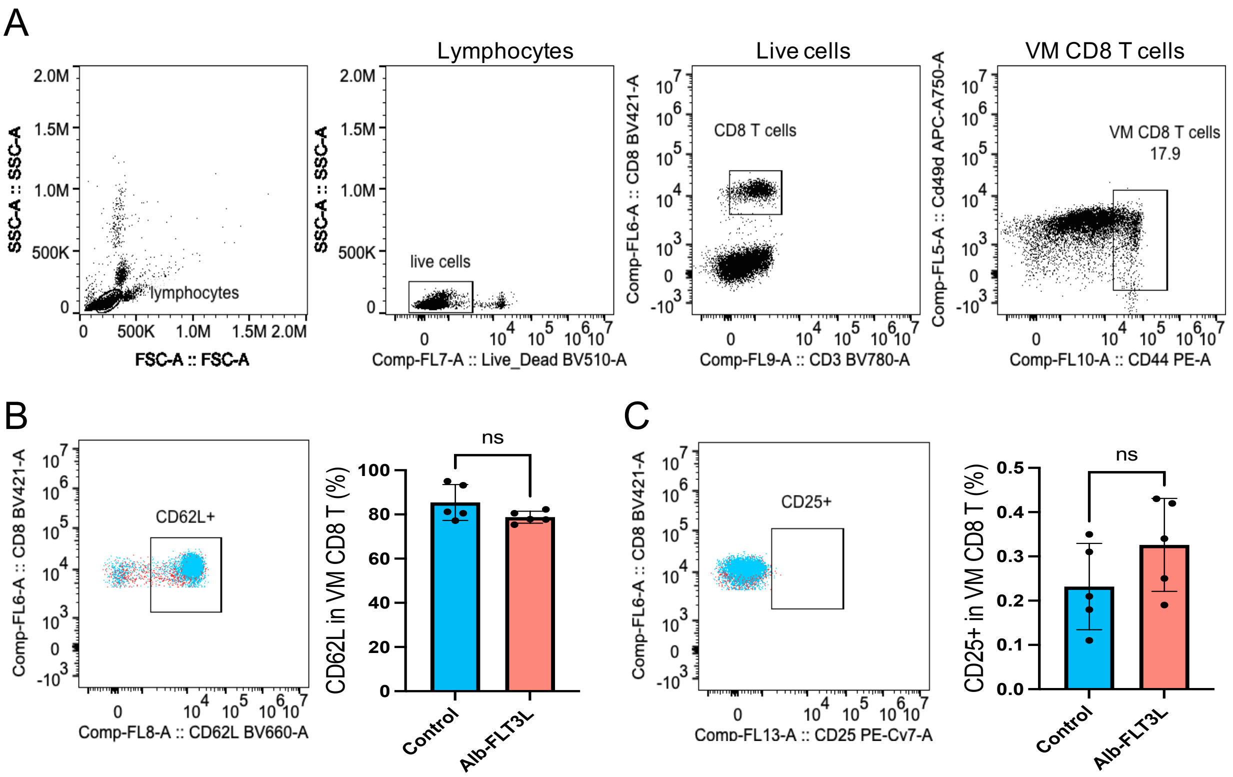

Figure S5. FLT3L-induced VM CD8 T cells were not been activated. C57BL/6 mice were administered either Alb-FLT3L (100 µg) or vehicle control on days 1, 5, and 8 for a total of 3 doses. PBMCs collected on Day 12 after initiation of treatment were assessed by flow cytometry. (A) Representative gating of VM CD8 T cells. (B) The frequencies of CD62L positive subsets in VM CD8 T cells. (C) The frequencies of CD25 positive subsets in VM CD8 T cells.

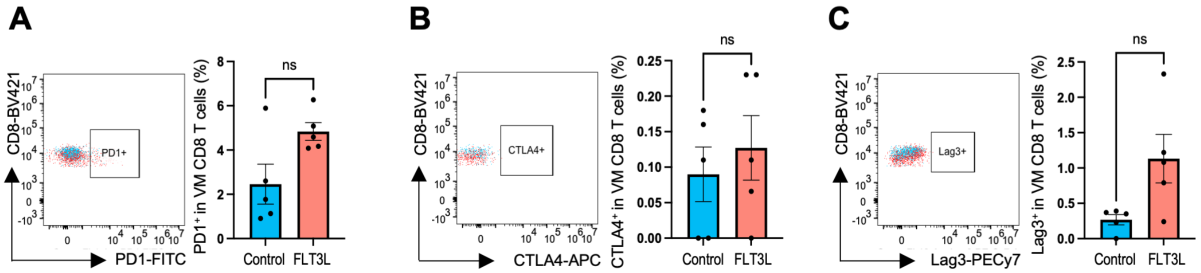

Figure S6. Expressions of exhaustion markers in VM CD8 T cells. C57BL/6 mice were administered either Alb-FLT3L (100 µg) or vehicle control on days 1, 5, and 8 for a total of 3 doses. PBMCs collected on Day 12 after initiation of treatment were assessed for exhaustion markers, including PD1, CTLA4, and Lag3, respectively. (A) The frequencies of PD1 positive subsets in CD8 T cells. (B) The frequencies of CTLA4 positive subsets in CD8 T cells. (C) The frequencies of Lag3 positive subsets in CD8 T cells.

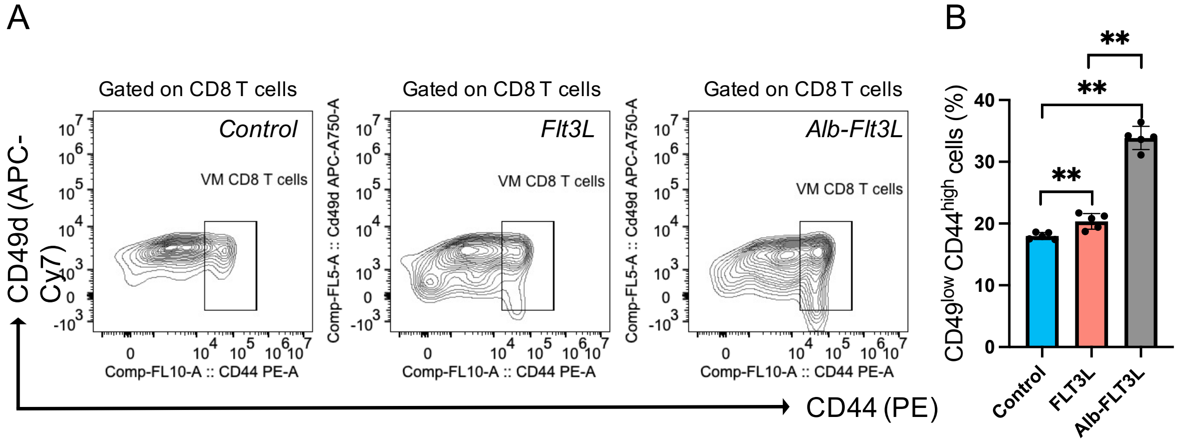

Figure S7. Frequencies of CD49^{low}CD44^{high}CD8 T cells in C57BL/6J mice receiving FLT3L and Alb-FLT3L treatment. C57BL/6 mice were administered either FLT3L (20 µg), Alb-Flt3L (100 µg) or vehicle control on days 1, 5, and 8 for a total of 3 doses. PBMCs collected on Day 12 after initiation of treatment were assessed by flow cytometry. (A) Representative gating of CD49^{low}CD44^{high} subsets on CD8 T cells (B) The frequencies of CD49^{low}CD44^{high} CD8 T cells for the indicated treatment groups.

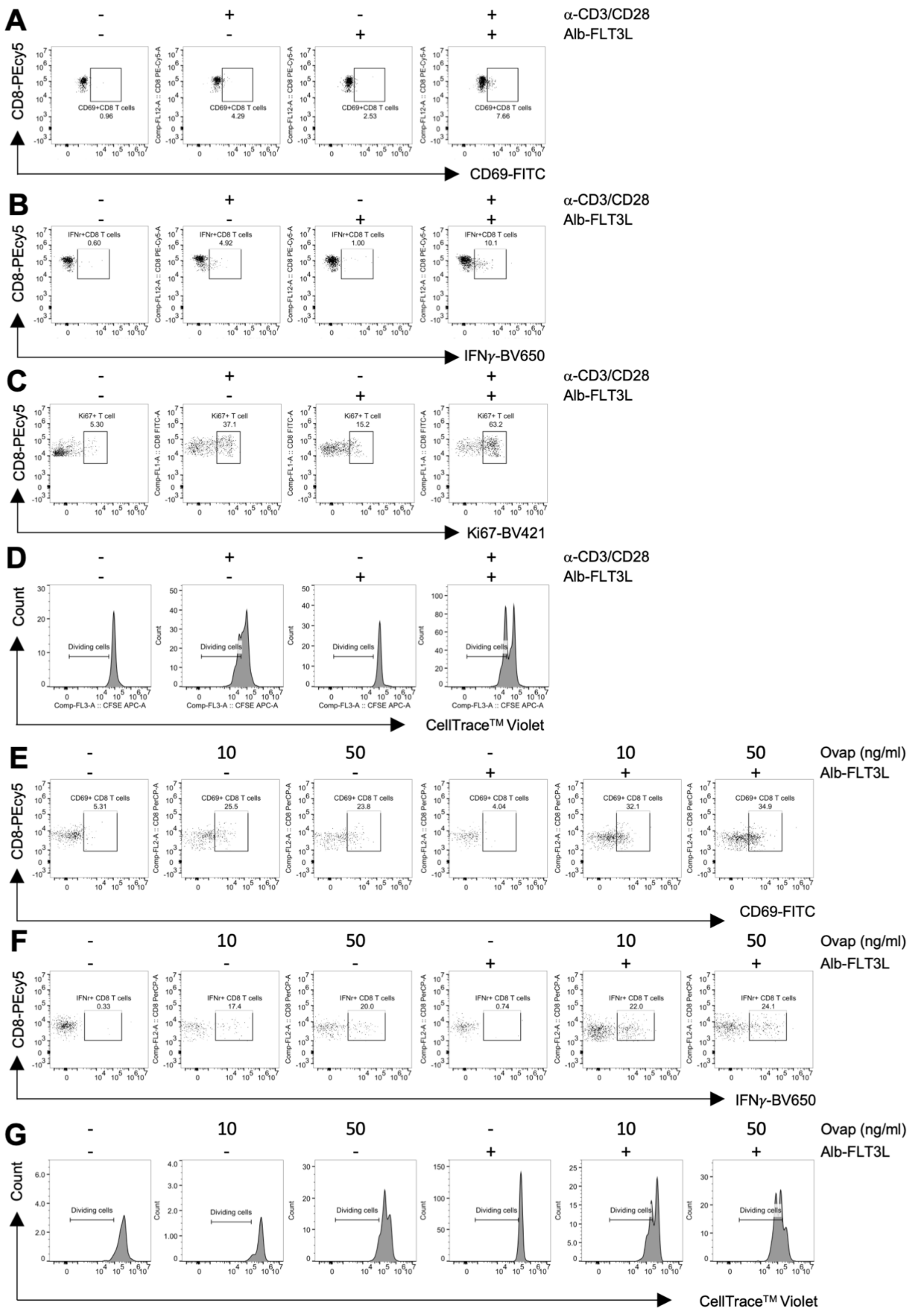

Figure S8. Representative figures for flow cytometry were shown in Figure 2.

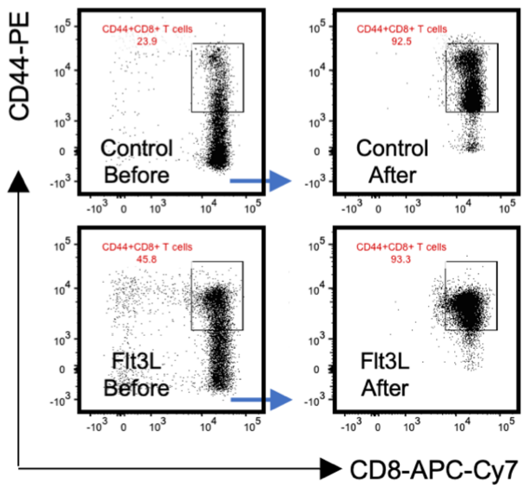

Figure S9. The FACS sorting strategy of CD44^high CD8 T cells from control naïve or FLT3L-treated OT1 mice.

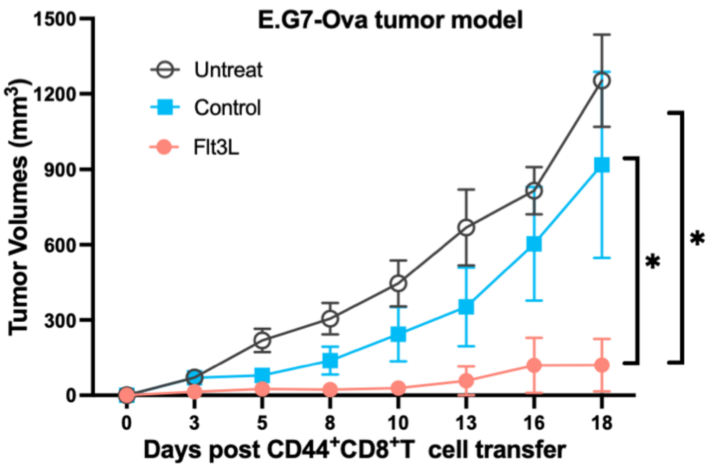

Figure S10. FLT3L-induced CD44^{high} CD8 T cells elicit tumor immunity against E.G7-ova tumors. C57BL/6 mice were inoculated with E.G7ova cells subcutaneously. Six days later, mice were injected intravenously through the retroorbital sinus with 2x10^{6} CD44^{high} CD8 T cells that were sorted from control naïve or FLT3L-treated OT1 mice. Eighteen days after adoptive T cell implantation, mice were euthanized. Tumor growth curve were recorded following the described treatment protocol.

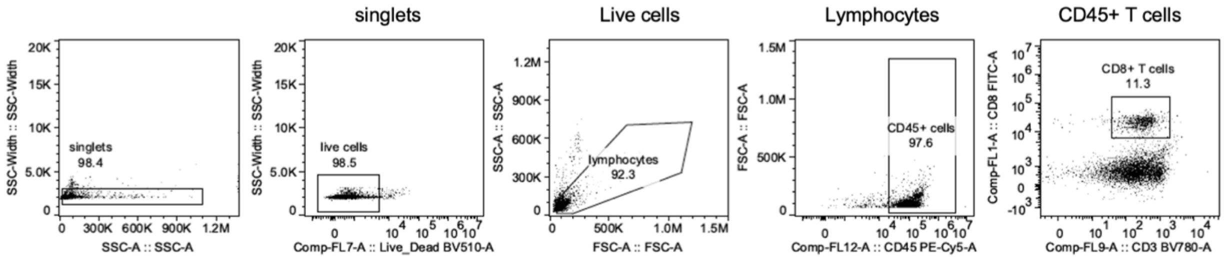

Figure S11. Gating strategies and representative figures for flow cytometry were shown in Figure 3C-3F.

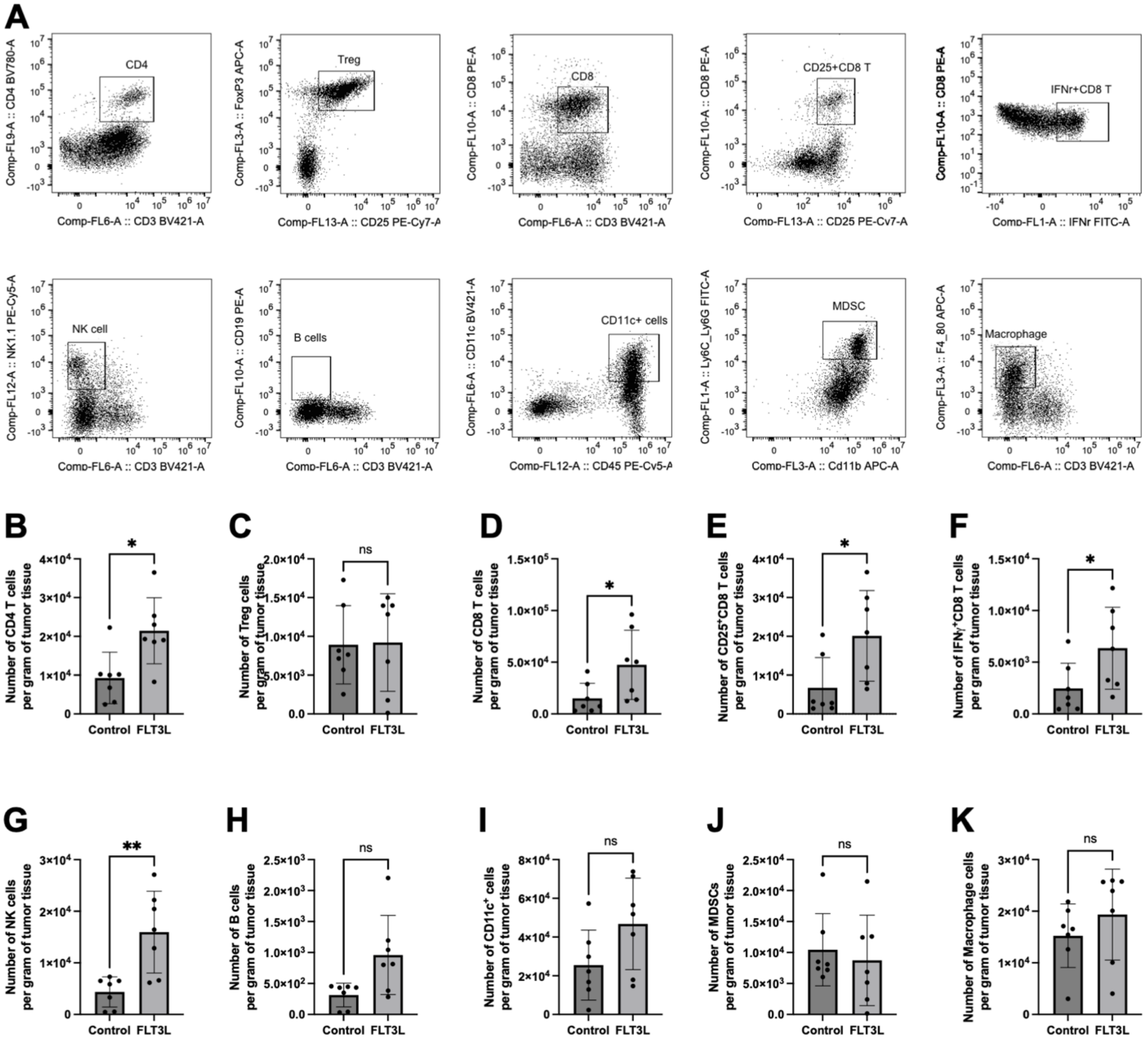

Figure S12. Tumor immunophenotype analysis in B16ova tumor-bearing mice received adoptive cell transfer of FLT3L-preconditioned CD44high CD8 T cells. C57BL/6 mice were inoculated with B16-OVA cells subcutaneously. Six days later, mice were injected intravenously through the retroorbital sinus with $2x10^6$ $CD44^{high}$ CD8 T cells that were sorted from control naïve or FLT3L-treated OT1 mice. Eighteen days after adoptive T cell implantation, mice were euthanized and tumor tissues were harvested for flow cytometry analysis. Cell numbers of immune subsets were normalized to individual tumor weights. (A) Representative figures for the gating strategy of immunophenotype analysis. (B-K) Lymphoid and myeloid immune subsets were quantified, including CD4 T cells, regulatory T cells, CD8 T cells, CD25+CD8 T cells, IFNγ+CD8 T cells, NK cells, B cells, CD11c+ DC cells, MDSCs, and macrophages, repectively.

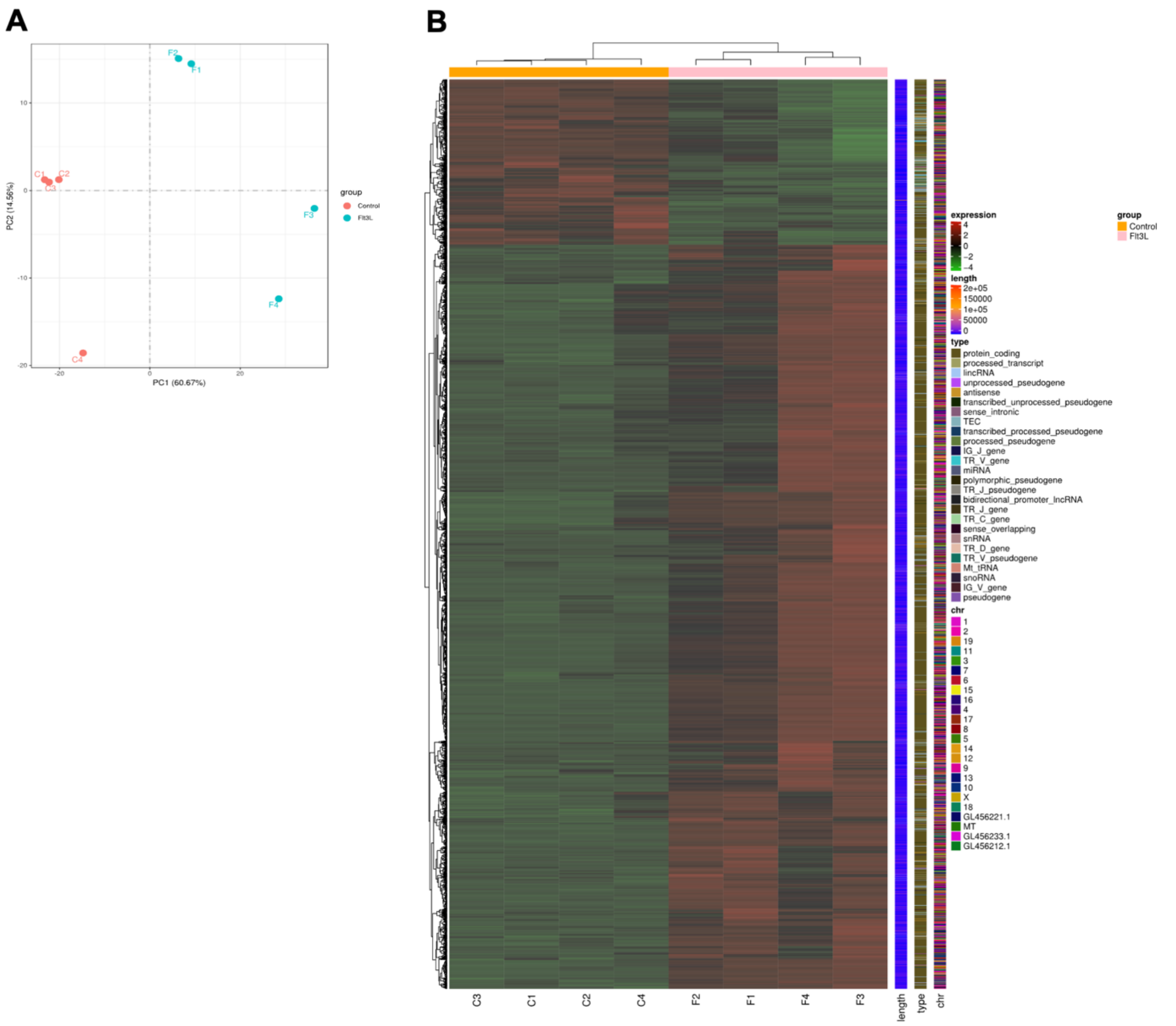

Figure S13. Bulk RNA-seq analysis of CD44^{high} CD8 T cells between FLT3L and control groups. (A) Principal component analysis (PCA) of differential genes of CD44^{high} CD8 T cells in control and FLT3L-treated mice. (B) Heat map of all the differential genes between control and FLT3L-induced CD44^{high} CD8 T cells.

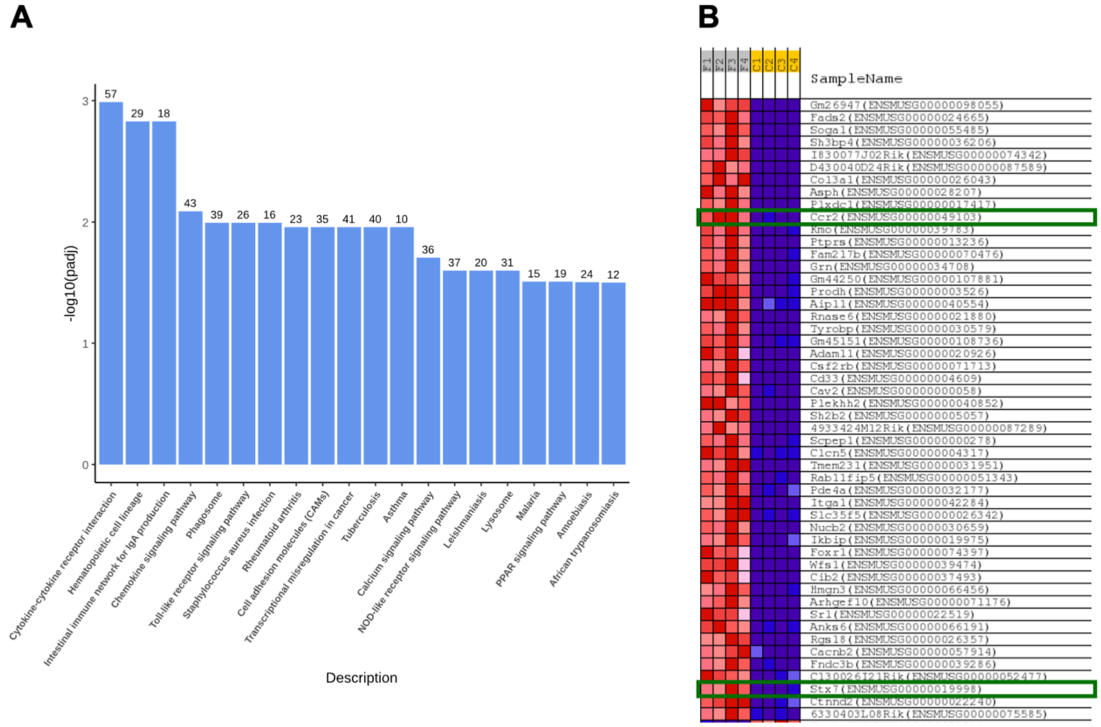

Figure S14. Differential gene expression analysis of bullk RNA-seq data. (A) Molecular function of GO enrichment analysis in genes that were up-regulated in FLT3L-induced CD44^{high} CD8 T cells. **(B)** Heat map of the top 50 upregulated differential genes between control and FLT3L-induced CD44^{high} CD8 T cells. Genes involved in inflammatory chemokine receptors and regulation of T cell activation and effector functions were highlighted in green.

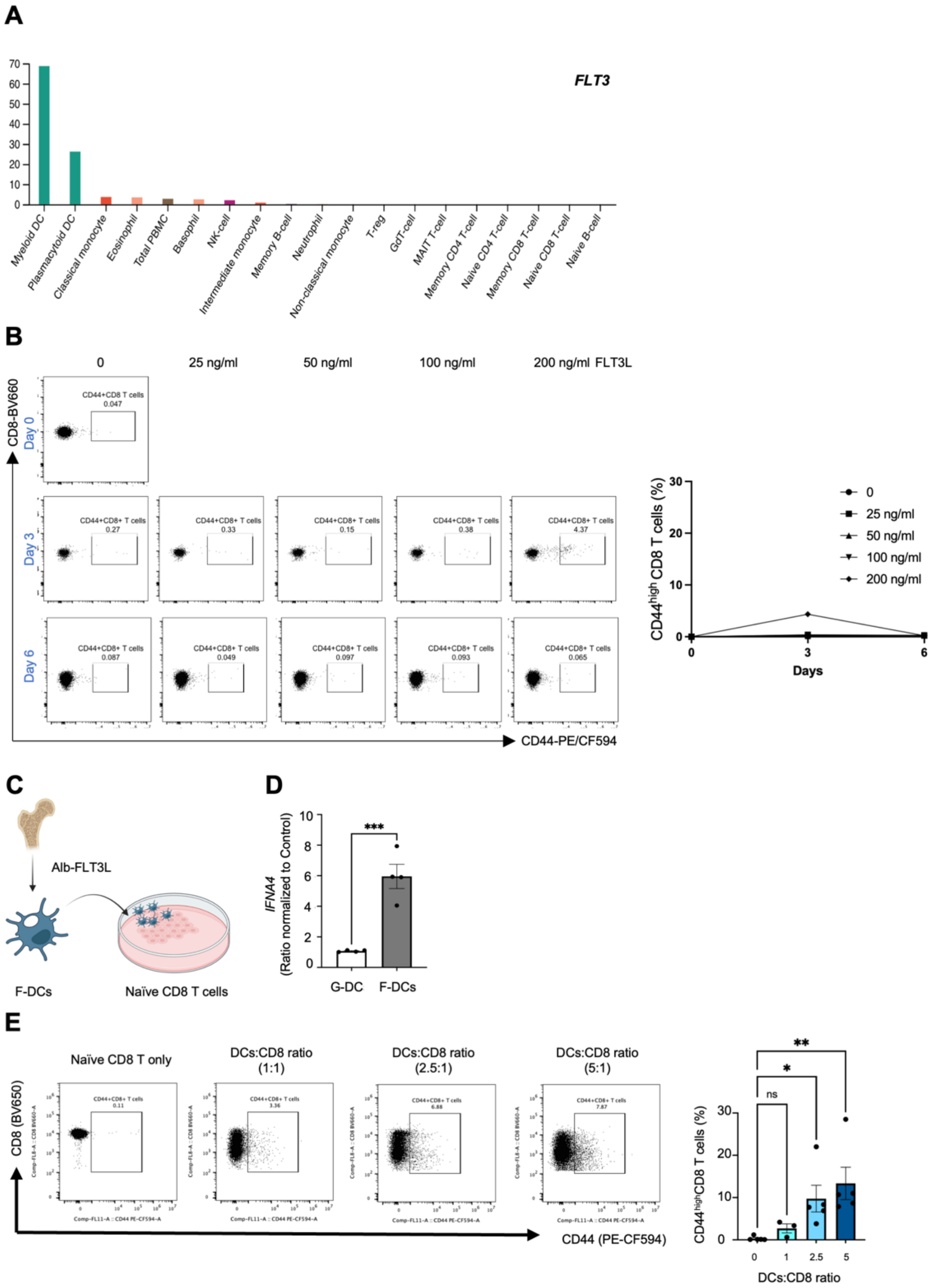

Figure S15. pDCs are critical mediators of FLT3L to drive CD8 T cells toward a virtual-memory phenotype. (A) Gene expressions of human FLT3 in immune cells from The Human Protein Atalas. (B) The naïve CD8 T cells were isolated by magnetic beads from 6-week-old C57BL/6 mice and incubated with indicated concentrations of FLT3L for 3 and 6 days. Representative gating of CD44 expressions on CD8 T cells in indicated days with different concentrations of FLT3L treatments. The frequencies of $CD44^{high}$ subsets in $CD3^{+}CD8^{+}$ T cells for

the indicated treatment groups. (C) The schematic graph represents co-culture Alb-Flt3L-derived BMDCs with Naïve CD8 T cells from C57BL/6 mice. (D) qPCR analysis of IFNa4 expression levels in either GM-CSF-induced or Alb-Flt3L-induced BMDCs. (E) Representative gating of CD44 expressions on CD8 T cells in the 3-day culture of T cells only and different rations of DCs-T cells of one representative experiment. Statistic of the frequencies of $CD44^{high}$ CD8 T cells for the indicated groups.

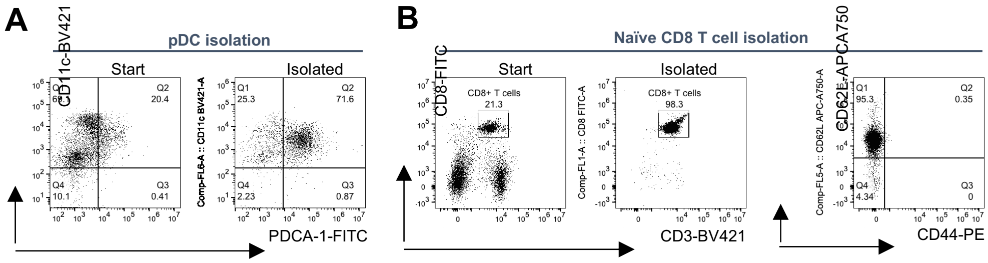

Figure S16. Magnetic isolation efficiency of pDC and naïve CD8 T cells. (A) Magnetic isolation efficiency of pDC (PDCA-1+CD11c+ cells) derived from the splenocytes of FLT3L-treated C57BL/6 mice. (B) Magnetic isolation efficiency of naïve CD8 T cells (CD3+CD8+CD62L+CD44- cells) derived from the lymphocytes and splenocytes of naïve C57BL/6 mice.

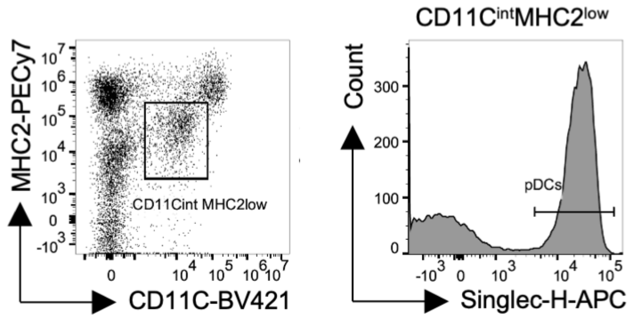

Figure S17. Gating strategies and representative figures of pDC for flow cytometry were shown in Figure 5A.

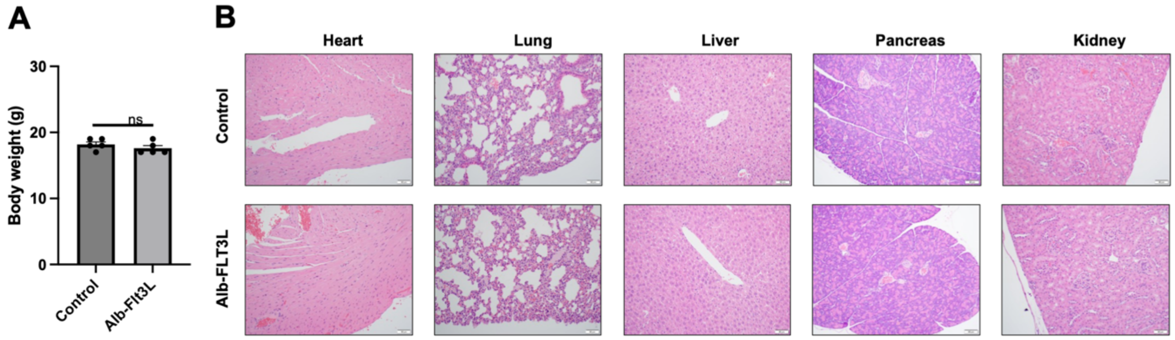

Figure S18. Body weight and H&E staining of major organs in C57BL/6 mice received Alb-FLT3L treatment. C57BL/6 mice were administered either Alb-FLT3L (100 ug) or vehicle control via i.p. injection for 12 days. Heart, lung, liver, pancreas, and kidney tissues were harvested and stored in 10% formalin prior to paraffin-fixed tissue embedding and sectioning. (A) Body weights were measured on Day 12 after Alb-FLT3L treatment. (B) Representative images of H&E stain of major organs from different treatment groups (20x).
